# Probing the extent of importin‐α targeting of the TAF8 NLS by eliminating its cationic net‐charge

**DOI:** 10.1002/pro.70272

**Published:** 2025-08-25

**Authors:** Amirabbas Abdoli, Zhihan Yang, Abdullah Odeh‐Ahmed, Olga Bednova, Bruno Lemieux, Leanne Dawe, Aymeric Ravel‐Chapuis, Pierre Lavigne, Natalie Zeytuni, Jeffrey V. Leyton

**Affiliations:** ^1^ Département de médecine nucléaire et radiobiologie, Faculté de médecine et sciences des sciences de la santé Université de Sherbrooke Sherbrooke Québec Canada; ^2^ Department of Anatomy and Cell Biology McGill University Montreal Québec Canada; ^3^ Centre de Recherche en Biologie Structurale (CRBS) Montreal Québec Canada; ^4^ Département de microbiologie et infectiologie, Faculté de médecine et sciences des sciences de la santé Université de Sherbrooke Sherbrooke Québec Canada; ^5^ School of Pharmaceutical Sciences, Faculty of Medicine University of Ottawa Ottawa Ontario Canada; ^6^ Department of Cellular and Molecular Medicine, Faculty of Medicine University of Ottawa Ottawa Ontario Canada; ^7^ Eric Poulin Centre for Neuromuscular Disease Ottawa Ontario Canada; ^8^ Département de pharmacologie, Faculté de médecine et sciences des sciences de la santé Université de Sherbrooke Sherbrooke Québec Canada

**Keywords:** importin‐α, microscale thermophoresis, NLS, nuclear transport, peptide engineering, TAF8, targeted therapeutics, zero net‐charge

## Abstract

The nucleus, as the control center of the eukaryotic cell, is a prime target for therapeutic interventions due to its role in regulating genetic material. Importin‐α is critical for successful nuclear import as it recognizes and binds to cargo proteins bearing a classical nuclear localization signal (NLS), which facilitates their transport from the cytoplasm into the nucleus. NLS tagging to ‘actively’ import therapeutics provides the most effective means of maximizing nuclear localization and therapeutic efficacy. However, traditional NLSs are highly cationic due to the recognition and binding requirements with importin‐α. Because of their highly ‘super‐charged’ nature, NLS‐tagged therapeutics face significant challenges, including poor pharmacokinetics due to non‐specific interactions. In this study, we engineered novel NLS tags with zero net charge to potentially overcome this limitation. Computational modeling and experimental validation revealed that these net‐neutral NLSs bind to importin‐α with similar modes and energies as their cationic counterpart. High‐resolution structural determination and analysis by X‐ray crystallography then confirmed their binding modes. Biophysical methods using circular dichroism, microscale thermophoresis, and cellular localization studies demonstrated that these NLSs maintain sufficiently stable complexes and acceptable binding to importin‐α and are functional. Additionally, this study revealed that the minor NLS‐binding site of importin‐α, with its extensive cationic surface area, was particularly suited for interactions with the acidic residues of the net‐neutral NLSs. This study provides a foundational understanding of NLS‐importin interactions and presents net‐neutral NLSs as viable candidates for next‐generation NLS‐therapeutic development and expands the scope of nuclear‐targeting therapies.

## 1 INTRODUCTION

Nuclear import relies on the presence of a nuclear localization signal (NLS) and its recognition by proteins known as importins (Goswami et al. 2024). Importins are a multigene family of soluble receptors that ferry NLS‐containing cargos in the cytoplasm across nuclear pore complexes (NPCs) and into the nucleus. Classical nuclear transport involves the initial binding of importin‐α to the NLS of cargo proteins in the cytoplasm. This is then followed by the association with importin‐β, which facilitates passage through the nuclear pore complex in a RanGTP‐dependent manner (Cingolani et al. 1999; Kobe 1999; Melchior et al. 1993; Moore and Blobel 1993).

Importin‐α possesses an α‐solenoid tertiary structure characterized by a deep concave surface ideal for NLS‐cargo binding (Fournier et al. 2013). Specifically, importin‐α consists of armadillo (ARM) domains, repetitive units of three α‐helices (termed H1, H2, and H3) arranged in tandem repeats (Fournier et al. 2013). Importin‐α consists of 10 ARM repeats forming an overall contorted cylindrical shape, with a ~30° right‐hand super‐helical twist (Kobe 1999). Notably, the twist causes the concave surface to become deeper at the N‐ and C‐termini, resulting in what is commonly known as the major (ARMs 2–4) and minor (ARMs 6–8) NLS‐binding grooves, respectively. They provide large surface areas, mostly negatively charged, that enable multivalent interactions with the elongated and positively charged NLSs.

Classical nuclear transport relies on NLSs that are categorized as monopartite or bipartite, based on the presence of one or two NLS motifs (Chang et al. 2013; Fontes et al. 2000; Zheng et al. 2018). Typically, monopartite NLSs can bind to both NLS‐binding grooves but prefer the major site, while bipartite NLSs require binding to both grooves for efficient nuclear transport (Chelsky et al. 1989; Fontes et al. 2000; Marfori et al. 2011; Marfori et al. 2012). Central to classical recognition are specific amino acids on an NLS that can be mapped to positions (P)1 through P6 within the major NLS‐binding groove (Goswami et al. 2024). Classical NLS models, exemplified by the ‘SV40’ large T‐antigen NLS (126‐PKKKRKV‐132) (Kalderon et al. 1984), emphasize NLSs need to be linearly elongated to fit well within the binding grooves. Additionally, the basic residue lysine is required at positions P2, while a lysine or arginine must bind at positions P3 and P5 (Conti and Kuriyan 2000; Fontes et al. 2000; Trowitzsch et al. 2015). As a result, the consensus of a classical monopartite NLS has been defined as K(K/R)x(K/R), with D/E/G being particularly disfavored at the P4 site (Chelsky et al. 1989; Hodel et al. 2001; Smith et al. 2018).

The nucleus being the control center of the cell, it is also the major susceptibility center and, hence, a desired target for drug designers (Bavelaar et al. 2018; Munsell et al. 2016; Shin et al. 2020). Drug targets include the DNA double helix and DNA‐interacting proteins (Bjornsti and Kaufmann 2019; Canavese et al. 2012; Martin et al. 2020). The deregulation of the nuclear import machinery is a key factor in many diseases (Hung and Link 2011; Mor et al. 2014), with increased importin gene expression often driving tumor cell proliferation and progression (Goswami et al. 2024). Therefore, understanding nuclear import principles, including their role in disease, offers potential avenues for advancing therapeutic strategies targeting the nucleus.

Because the NPC is a permeability barrier and limits the passage of macromolecules, it is also a formidable blockade for therapeutic nuclear access. ‘Active’ nuclear‐directed import and efficient translocations across NPCs are crucial for maximizing nuclear localization (Goswami et al. 2024). Despite advancements in NLS‐based nuclear targeting systems, additional insight is needed to elucidate the complexities of nuclear transport, disease cellular contexts, and pharmaceutical aspects like the mode of administration.

We previously developed diverse NLS‐tagged antibody‐conjugate therapeutics targeting multiple tumor types, demonstrating their ability to eradicate cancer cells in vivo (Fasih et al. 2012; Leyton et al. 2011; Leyton et al. 2015; Paquette et al. 2018). Upon intravenous administration, the NLS‐antibody conjugates cleared significantly faster than the unmodified antibody counterparts (Fasih et al. 2012; Paquette et al. 2018). This was most likely caused by the highly cationic net charge (5+) of the SV40 NLS. Studies have shown that antibody therapeutics with increased net‐positive molecular charge have poor pharmacokinetics due to increased non‐specific ionic interactions with cell membranes that, in part, increase the rate of degradation (Datta‐Mannan et al. 2015; Hinton et al. 2006; Igawa et al. 2010; Li et al. 2014). Tagging methods typically append 10–20 NLSs per antibody (Beaudoin et al. 2016; Chen et al. 2006), making NLS‐therapeutics ‘super‐charged’ cationic agents with limited pharmacokinetics.

The general transcription factor TFIID is essential for RNA polymerase II transcription initiation in eukaryotic cells. This megadalton protein complex, composed of several TATA‐binding‐associated factors (TAFs), is assembled into a functional transcription factor in the nucleus (Thomas and Chiang 2006). RNA polymerase II and its associated transcription factors are upregulated in cancer (Sadurni 2023). The Berger group recently elucidated how an essential TFIID subcomplex consisting of TAF2, TAF8, and TAF10 is assembled in the cytoplasm (Trowitzsch et al. 2015). Using a peptide containing TAF8's amino acid sequence 297‐PVKKPKIRRKKSLS‐310, its structure bound to importin‐α was solved, revealing the PVKKPKIRR sequence bound the major groove, while only the KKS sequence bound the minor groove, which indicated that while the sequence most likely independently sampled both binding grooves, only the PVKKPKIRR sequence bound preferentially at the major groove. Silencing TAF8 expression prevented TAF2 and TAF10 from localizing in the nucleus. Isothermal titration calorimetry (ITC) studies of TAF8 residues 297–310 determined a dissociation constant (*K*_D_) of 10.4 ± 0.8 μM, supporting a 1:1 stoichiometry and further suggesting that the PVKKPKIRR sequence constituted the functional TAF8 NLS and that it was major groove specific.

Published crystal structures and theoretical energy estimations suggest that the molecular recognition of an NLS sequence by importin‐α in the major binding groove has an estimated free energy of at least Δ*G* ~ −10 kcal/mol (Delfing et al. 2023; Hodel et al. 2001). The P2 site interactions are dominant and the energetic contributions of the P3–P5 range from 25%–66% of the P2 site contribution, with the P4 site interactions contributing the lowest among the consensus (Delfing et al. 2023). The amino acids that bind at the P1 and P6 sites confer shape to the NLS bound pose within the pocket, which contributes to specificity for different importin‐α subtypes (Fontes et al. 2000) or minor versus major groove preferences (Chang et al. 2013). The Klimov group recently showed that the minimum sequence of KKPK matching exactly TAF8 NLS P2–P5 binding residues, transitioned between diverse binding poses due to multiple free energy states in the major binding groove favorable for binding (Delfing et al. 2023). Interestingly, adding a C‐terminal glutamic acid significantly strengthened binding to importin‐α. Therefore, targeting the nucleus by exploiting the increased nuclear import associated with overexpressed RNA polymerase II and/or its associated transcription factors and the discovery that acidic amino acids potentially play a role in TAF8 NLS binding to importin‐α provides an opportunity for NLS‐therapeutic development by specifically reducing its highly cationic nature.

However, nuclear import is an intricate process that must be carefully considered. Nuclear import rates correlate with the formation of the importin‐α/β/NLS‐cargo complex in the cytoplasm (Timney et al. 2006). Additionally, nuclear import kinetics are influenced by the cytoplasmic concentrations of both NLS‐cargo proteins and importin‐α, as well as NLS affinity. It is tempting to speculate that increased NLS affinity will proportionally result in improved therapeutic efficacy. However, to the best of our knowledge, this has never been demonstrated and underscores the deep complexity of NLS‐therapeutic development. Therefore, the early design stages of potential NLS candidates with the net cationic charge eliminated must maintain comparable interaction strengths and binding modes to importin‐α, and nuclear localization as the natural NLS counterpart to minimize any potential disturbance of the common thermodynamics and kinetics of nuclear import.

In this study, we describe a combined computational and biophysical approach to design TAF8 NLS analogs, with zero net charge, and test their ability to target importin‐α. The analogs were not explicitly designed to bind with higher affinity but to have comparable interaction strengths and binding modes. To validate the computational modeling design, the analogs were co‐crystallized with importin‐α, and their structures were experimentally determined by X‐ray crystallography. Circular dichroism (CD) and microscale thermophoresis (MST) assays were performed to quantify these interactions and calculate their stoichiometry, complexation stabilities, and *K*_D_ values. Cellular studies were performed to evaluate nuclear localization. Our results reveal that it is possible to develop NLS analogs with a zero net charge based on TAF8 NLS that still maintain comparable binding strength and modes and can localize to the nucleus. Additionally, potential alternative amino acid interactions are discovered, as well as minor groove binding preference that provides avenues for future synthetic NLS development and/or searching for rare NLS types in nature.

## 2 RESULTS

### 2.1 TAF8 NLS binding mode characteristics in the major groove of importin‐α

A visual and interaction energy‐based inspection of TAF8 NLS–importin‐α complex (Trowitzsch et al. 2015) was performed to identify suitable positions within the NLS sequence to switch to generate zero net‐charged analogs. TAF8 NLS binds to the major groove with a total contact energy of −76.2 kcal/mol. K299, K300, and K302 provide 87.9% of the total NLS binding energy, with K299 and K302 making the strongest energetic interactions (Table 1). Apart from the previously identified contacts made by K299 with the side chains D192, G150, and T155 (Trowitzsch et al. 2015), several Van der Waals (VdW) force interactions are made with residues A148, S149, T151, S152, N188, G191, and W231. K300 forms hydrogen bonds with N188 and N228, cation–π and hydrogen bonds with W184, and VdW force interactions with S149 and W231.

**TABLE 1:** Contact energy distributions of the crystal structure analogs complexed to the major NLS‐binding groove of importin‐α.

NLS (net‐charge)	Individual energetic contribution per residue (%)									Total (kcal/mol)
	297	298	299	300	301	302	303	304	305	
TAF8^a^ (5+)	P (7.6)	V (7.7)	K (31.7)	K (22.7)	P (1.1)	K (33.5)	I (2.4)	R (^b^)	R (0)	−76.2
A1 (0)	E (4.7)	V (5.5)	K (25.9)	K (27.0)	P (0.9)	K (26.6)	I (5.0)	D (2.1)	E (2.5)	−74.93
A2^c^ (0)	P (31.3)	V (333.5)	K (−78.5)	K (−88.4)	E (−5.7)	K (−69.7)	I (−12)	E (−7.0)	E (−3.9)	28.24
A3 (0)	P (0)	E (2.2)	K (38.0)	K (23.3)	P (1.1)	K (29.3)	I (3.7)	D^d^ (0)	E (3.3)	−67.67
A4 (0)	P (3.8)	V (6.5)	K (32.7)	K (26.5)	E (^b^)	K (26.9)	I (5.1)	D (2.3)	E (1.9)	−75.46

^a^ TAF8 NLS is complexed to human importin‐α1 (Trowitzsch et al. 2015), while the analog structures were determined complexed to murine importin‐α2.

^b^ Amino acid interactions clashed with importin‐α side chains and positive contact energies. These energies were included in the total contact energy for TAF8 NLS and analogs but are not calculated in the % individual energetic contribution per residue.

^c^ A2 had an overall positive contact energy. Positive and negative percentages reflect contact energies that contributed to repulsive and favorable binding forces, respectively.

^d^ Based on the in silico design and the significant amount of contact energy contributed by E304 for C3, it was changed for an aspartic acid residue.

**FIGURE 1:**
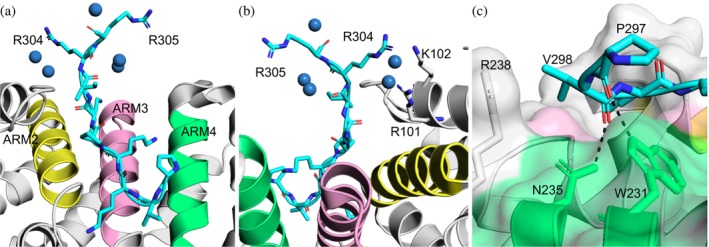
C‐terminal arginine and N‐terminal proline binding mode of TAF8 NLS. (a) Top and (b) bottom views of TAF8 NLS (cyan) in the major groove defined by the H3 helices of ARMs 2, 3, and 4 colored yellow, pink, and lime, respectively, of human importin‐α1. The five waters that stabilize the R304 and R305 at the C‐terminus are shown as blue spheres. (c) Surface rendering showing the positioning of P297 and V298 of TAF8 NLS at the P1 site of the major groove. Dashed lines depict the hydrogen bonds between P297 and W231 and V298 and N235. V298 also forms a VdW interaction with R238. The colors indicate the surfaces for the H3 helices of ARMs 2–4, as indicated above. Adapted from PDB: 4WV6 (Trowitzsch et al. 2015).

The TAF8 NLS residues R304 and R305, at the C‐terminus, are stabilized by water molecules in the crystal cell (space group P2_1_2_1_2_1_). Upon deeper inspection, R304 and R305 form contacts with five water molecules (Figure 1a). As a result, R304 and R305 are oriented up and out of the major groove (Figure 1b). R305 does not engage with any importin‐α side chains, while R304 sterically clashes with side chains R101 and K102 (Figure 1b) in ARM1 and suggests that both arginine residues are mobile, can possibly conform to additional configurations, and are suitable for substituting with acidic amino acids.

A common but less understood feature of NLSs, which is the N‐terminal proline, was also evaluated. Several NLS sequences contain N‐terminal prolines that bind at the P1 site of the major NLS‐binding groove (several are listed in de Oliveira et al. 2021) and influence binding affinity. The P1 site for human importin‐α is characterized by a small valley‐shaped‐like pocket with sharp rising peaks composed of W231 and R238 on the N‐ and C‐terminal sides, respectively (Figure 1c). R238 rises sharply from the P1 site floor and points inwards towards the NLS‐binding groove, while the indole of W231 lies sideways, forming a broad shallow peak. R238 comprises a portion of the hinge that connects ARMs 4 and 5 and defines the C‐terminal boundary of the major groove (Kelley et al. 2010), while W231 is part of the H3 helix of ARM4.

V298 of TAF8 NLS forms a hydrogen bond and VdW interaction with N235 and R238, respectively. Importantly, the interaction of R238 and V298 prevents P297 from extending further outside the major groove and results in P297 forming a ‘hairpin‐turn,’ common to NLSs with N‐terminal proline residues, which is stabilized by a hydrogen bond with W231. The C‐terminal carboxyl group of P297 forms the hydrogen bond with the nitrogen atom in the indole side chain of W231. As a result, the pyrrolidine side chain “floats out” of the NLS‐binding pocket and does not make any contacts (Figure 1c). To further support this, other NLSs with this N‐terminal proline motif, such as SV40 and c‐Myc, which also have the hairpin‐turn binding modes (Conti and Kuriyan 2000; Fontes et al. 2000), were evaluated (Figure S1, Supporting Information). These NLSs bind to importin‐α from different species (TAF8:human, SV40:mouse, and c‐Myc:yeast) and were modeled onto the mouse importin‐α paralog 2 (mImpα2) as it is almost identical to its human importin‐α1 counterpart in amino acid sequence similarity (Delfing et al. 2023), which was subsequently used for crystallization experiments. P297 and V298 side chains, including the modeled counterparts, were not involved in hydrogen bonds or salt bridge formations with the side chains of importin‐α (Figure S1), opening the plausibility to introduce mutations to generate zero net‐charge analogs that can also interact at the P1 site.

### 2.2 In silico design of TAF8 NLS‐based zero net‐charge analogs

The in silico design of TAF8 NLS‐based zero net‐charge analogs resulted in the successful creation of four variants (A1–A4) maintaining statistically significant comparable docking scores and binding modes to TAF8 NLS within the major NLS‐binding groove of human importin‐α. As anticipated, alanine scanning highlighted the sensitivity of amino acids K299, K300, and K302 at the P2, P3, and P5 sites. Substituting K299 for an alanine residue impaired the overall NLS binding up to 10% (Figure S2a). In contrast, alanine residue substitutions at positions P297, V298, P301, and I202 resulted in ≤2% reduced docking scores, while there was an improvement for R304 (Figure S2a).

The subsequent substitutions of negatively charged residues at the P1, P4, and P6 sites revealed that R305, protruding outside the major binding groove, could be substituted with glutamic or aspartic acid. The most impaired docking, a 3.5% reduced score, was with a glutamic acid substitution at I303, marking our threshold for acceptable loss in binding strength. Acidic residue substitutions at the other positions resulted in ≤3.0% reduction, allowing for diverse combinations of zero net‐charge analogs at sites P297, V298, and R304 for testing.

**FIGURE 2:**
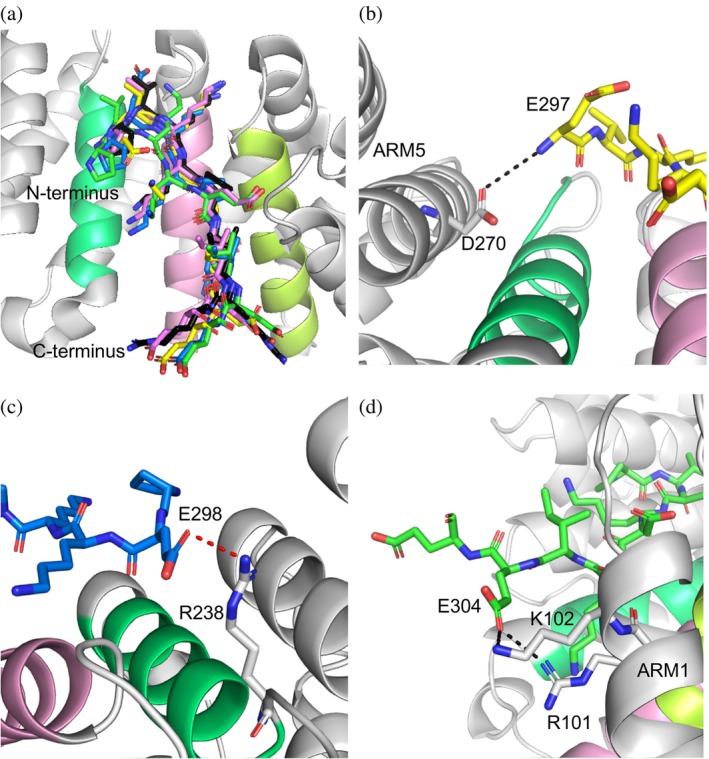
Novel contacts for the modeled zero net‐charge analogs. The analogs (A1: yellow, A2: green, A3: marine, A4: magenta) formed a network of contacts with amino acid side chains that are outside the major NLS‐binding pocket of human importin‐α1. (a) Analog binding modes at the major groove. Black NLS is the TAF8 NLS pose from the resolved crystal structure (PDB: 4WV6; Trowitzsch et al. 2015). ARMs 2–4 are colored as in Figure 1. (b) E297 of A1 formed a hydrogen bond (dashed black line) with D270. (c) E298 of A3 formed a salt bridge (dashed red line) with R238. (d) E304 of A2 forms two hydrogen bonds with R101 and K102 and an additional salt bridge (no line shown) with K102.

Figure S2b lists the frequency of distribution of the 100 docked poses of A1–A4 with statistically significant docking scores comparable to the in silico version of TAF8 NLS. A3 exhibited the best frequency of distribution with superior median (−28.09 kcal/mol) and mean (−28.10 ± 7.33 kcal/mol) scores, suggesting it formed the most stable interactions with the major groove. Additionally, the binding modes for A1–A4 were very similar to one another and the crystal structure of TAF8 NLS (Figure 2a).

### 2.3 Zero net‐charge analogs form novel interactions with human importin‐α

Table 2 lists the contact energy distribution of TAF8 NLS and the zero net‐charge analog top poses docked at the major groove of human importin‐α. The analogs made 6–8 hydrogen bonds, 1–4 salt bridges, 29–33 VdW interactions, and 1–2 cation–π bonds. In comparison, the top pose TAF8 NLS made 7 hydrogen bonds, 2 salt bridges, 33 VdW interactions, and 1 cation–π bond.

**TABLE 2:** Contact energy distribution of top docked poses for TAF8 NLS and the zero net‐charge analogs at the major NLS‐binding groove of human importin‐α.

NLS	Individual energetic contribution per residue (%)									Total (kcal/mol)
TAF8	P (0)	V (16.7)	K (31.4)	K (42.6)	P (0)	K (13.4)	I (2.7)	R (^a^)	R (0)	−51.1
A1	E (4.0)	V (18.8)	K (40.1)	K (20.8)	P (^a^)	K (19.4)	I (0)	D (0)	E (0)	−38.44
A2	P (0)	V (4.2)	K (8.9)	K (9.7)	E (4.3)	K (39.0)	I (3.3)	E (30.5)	E (0)	−54.15
A3	P (0)	E (26.9)	K (34.7)	K (7.3)	P (^a^)	K (11.4)	I (3.4)	E (17.7)	E (0)	−49.3
A4	P (0)	V (14.1)	K (49.2)	K (10.5)	E (^a^)	K (26.3)	I (3.1)	D (0.6)	E (0)	−45.2

^a^ Amino acid interactions clashed with importin‐α side chains and positive contact energies. These energies were included in the total contact energy for TAF8 NLS and analogs but cannot be calculated in the % individual energetic contribution per residue.

Notably, the N‐terminal amine of E297 of A1 formed a hydrogen bond with the side chain of D270, which is located outside the binding groove on ARM5 but in the vicinity of the P1 site (Figure 2b). For A3, the side chain of E298 formed extensive bonds with nearby amino acids of importin‐α and contributed the second highest amount of contact energy, behind K299 (Table 2). E298 formed a novel salt bridge with R238 at the C‐terminal edge of the P1 site (Figure 2c), while maintaining the important hydrogen bonds with the side chains of W231 and N235 deep in the binding groove. For A2 and A3, the side chain of E304 extended out of the major groove as was the case for R304 of TAF8 NLS (Figure 1a,b). However, E304 formed multiple new hydrogen bonds and salt bridges with R101 and K102 in ARM 1 (Figure 2d), whereas the natural R304 previously clashed with these residues (Figure 1b).

Taken together, in silico design of potential NLS analogs with zero net charge derived from TAF8 NLS is possible for at least three reasons. One, the C‐terminal R305 residue does not play a role in binding importin‐α and can be substituted for an acidic residue. Two, glutamic acid substitutions at positions P297, V298, and E304 can form novel contacts with amino acid side chains at the edges (at the P1 and P6 sites) of the major NLS‐binding groove. Three, the A1–A4 analogs, with the diverse interspersed acidic amino acids, can bind the major groove with comparable docking scores and maintain very similar binding modes compared to TAF8 NLS.

### 2.4 Atomic structures of the four zero net‐charge analogs bound to mImpα2 and a comparative analysis between the crystallized and modeled structures

**FIGURE 3:**
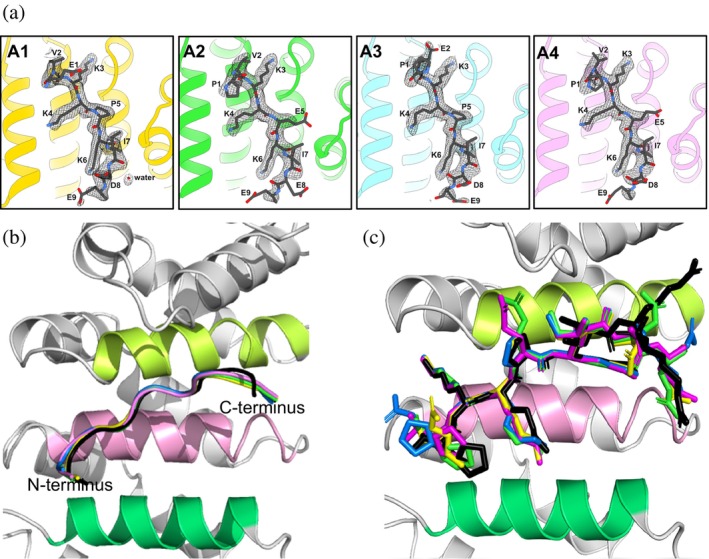
Resolved crystal structures of the zero net‐charge analogs bound to the major groove of mImpα2. (a) The Fo–Fc difference maps of A1–A4 at the major groove of mImpα2. The superposition of the (b) Cα's and (c) side chains of the determined crystal structures of A1 (yellow), A2 (green), A3 (marine), and A4 (magenta) in the major groove of mImpα2, along with TAF8 NLS (black) from PDB: 4WV6 (Trowitzsch et al. 2015).

The crystal structures of A1–A4 in complex with mImpα2 at resolutions ranging from 2.11 to 2.42 Å were determined (Table 3). The electron densities of the 10 ARM domains of mImpα2 without the importin‐β‐binding (IBB)‐domain were clearly resolved including the P1–P6 and P′1–P′5 sites for the major and minor NLS‐binding grooves, respectively. The crystal structures had the same space groups as the original TAF8 NLS/importin‐α complex and every amino acid was resolved for all analogs bound at the major groove (Figure 3a). Accordingly, the analogs bury an average surface area of 629.6 ± 21.2 Å^2^ in the major groove.

**TABLE 3:** Data refinement statistics of the crystals.

NLS Analogs	A1	A2	A3	A4
PDB code	9CL8	9CLE	9CLF	9CLG
Space group	P2_1_2_1_2_1_	P2_1_2_1_2_1_	P2_1_2_1_2_1_	P2_1_2_1_2_1_
Molecules/unit	1	1	1	1
Cell dimensions				
*a*, *b*, *c* (Å)	78.41, 89.70, 99.42	78.43, 89.82, 99.66	78.40, 89.75, 99.52	78.01, 89.96, 99.16
*α*, *β*, *γ* (°)	90, 90, 90	90, 90, 90	90, 90, 90	90, 90, 90
Resolution (Å)	50–2.11	50–2.34	50–2.36	50–2.42
*R*_merge_ (%)	7.3 (50.1)	8.0 (54.1)	9.2 (55.8)	7.2 (48.3)
*I*/*σI*	21.8 (2.5)	20.67 (2.58)	18.65 (2.37)	20.37 (2.71)
Completeness (%)	100 (100)	100 (100)	100 (100)	99.9 (99.8)
Redundancy	6.6 (6.9)	6.4 (6.6)	6.4 (6.6)	6.4 (6.3)
CC1/2	0.999 (0.901)	0.997 (0.862)	0.995 (0.870)	0.997 (0.928)
CC*	1.000 (0.974)	0.999 (0.962)	0.999 (0.964)	0.999 (0.981)
Wavelength (Å)	0.9537	0.9537	0.9537	0.9537
Refinement				
Resolution (Å)	49.761–2.11	49.880–2.34	49.811–2.36	49.628–2.42
No. reflections (unique)	41,131	30,438	29,674	27,207
*R*_work_/*R*_free_ (%)	15.95/19.16	15.72/20.50	15.64/20.78	16.07/20.24
No. of atoms				
mImpɑ2	3285	3287	3288	3265
Ligand/ion	32	58	34	52
Water	313	260	246	190
NLS analogs (major groove)	75	76	75	75
NLS analogs (minor groove)	9	41	50	50
B‐factors (Å)				
mImpɑ2	42.47	45.65	46.34	47.17
Ligand/ion	70.08	79.75	81.6	79.20
Water	47.83	49.26	47.32	48.66
NLS analogs (major groove)	59.26	73.32	73.56	74.02
NLS analogs (minor groove)	81.47	108.51	117.36	110.486
MolProbity score	1.21	1.34	1.44	1.20
Rotamer outliers (%)	0.27	1.32	1.58	0.8
Ramachandran favored (%)	98.84	97.92	98.16	98.39
Ramachandran allowed (%)	1.16	2.08	1.84	1.61
Ramachandran outliers (%)	0	0.23	0.23	0.23
RMS deviations				
Bond lengths (Å)	0.0090	0.0071	0.0070	0.0074
Bond angles (°)	1.5319	1.3672	1.4036	1.4706
Buffer condition	0.8M NaCit, 0.1M HEPES pH 6.4, 60% glycerol, 10 mM DTT	0.7M NaCit, 0.1M HEPES pH 6.6, 60% glycerol, 10 mM DTT	0.65M NaCit, 0.1M HEPES pH 7.0, 60% glycerol, 10 mM DTT	0.8M NaCit, 0.1M HEPES pH 6.2, 60% glycerol, 10 mM DTT

*Note*: Statistic values present in parentheses correspond to the highest resolution shell data.

Superpositioning the four analog structures at the major groove showed that the sequences also share almost identical binding modes, based on the alpha‐carbon's (Cα's) as the crystal structure of TAF8 NLS (Figure 3b). The Cα backbone of the analogs exhibited RMSD values of 0.485, 0.529, 0.487, and 0.513 Å for A1, A2, A3, and A4, respectively, at the major groove.

Interestingly, the analog side chains displayed diverse avidities, all markedly different from TAF8 NLS. In the published structure of human importin‐ɑ bound to TAF8 NLS, the resolved number of atoms at the major and minor NLS‐binding grooves was 3,264 for importin‐ɑ, 78 for the major groove ligand, and 24 for the minor groove ligand (Trowitzsch et al. 2015). The analog crystal structures had highly similar atom counts (Table 3). The B‐factors, which reflect atomic mobility or flexibility in a crystal structure,  were lower for human importin‐ɑ1 and both bound ligands compared to the analogs and their corresponding mImpɑ2 complexes (Table 3). Among the analogs, the A1/mImpα2 complex exhibited the greatest stability based on B‐factors. The analogs formed a total of 8–10 hydrogen bonds, 1 salt bridge, 37–40 VdW interactions, and 1–2 cation–π bonds. In comparison, TAF8 NLS formed 11 hydrogen bonds, 1 salt bridge, 35 VdW interactions, and 2 cation–π bonds. As the total number and types of bonds formed by the analogs were similar to TAF8 NLS, it was not apparent why the analogs had varying B‐factors that were notably higher and reflecting less stability. Most likely, subtle yet vital contacts account for these differences.

Visual inspection revealed that although the side chains for K299, K300, and K301 residues aligned at the P2, P3, and P4 sites, indicating that the analogs also relied on the consensus sequences for their binding (Figure 3c), there were notable spatial orientational differences for some other residues and the amino acid contributions for A1–A4 (Table 1) changed significantly from the computational modeling (Table 2). For example, the P297 residues of each analog were positioned differently from each other and from TAF8 NLS (Figure 3c). A1, the only analog with an N‐terminal glutamic acid, was positioned over W231, matching the spatial position of P297 of TAF8 NLS and the hydrogen bond formed with W231 (Figure S3a vs. Figure 2b). However, instead of forming a hydrogen bond with the side chain of D270, a hydrogen bond was formed with the solvent molecule DTT, derived from the crystallization buffer (Figure S3a). E297 was further stabilized by forming hydrogen bonds with two water molecules. DTT also interfered with the N‐terminal P297 of A3. Instead of forming a bond, DTT had an energetically repulsive effect on P297 and pushed it into the binding groove (Figure S3b). As a result, E298 was shifted too far away to form a salt bridge with the side chain of R238 (Figure S3b vs. Figure 2c) and was stabilized by a hydrogen bond with a water molecule. As previously mentioned, it was not an aim to generate zero net‐charged NLSs with superior binding affinity, as it is unknown how this would affect natural classical nuclear transport. Hence, E304 of A3 was changed to D304 as the former contributed 17.7% of the total contact energy (Table 2). D304 in the crystal structure of A3 complexed to mImpα2 was not able to form bonds with R101 and K102, previously observed with E304 (Figure S3c vs. Figure 2d). However, E304 of A2 was also unable to form bonds with R101 and K102 and, again, was due to DTT interference (Figure S3c). DTT was slotted near the P1 site. An energetic analysis revealed that DTT formed a hydrogen bond with the pyyrolidine amine of P297. As a result, P297 was not able to position itself further up the N‐terminus of the H3 helix of ARM4, as observed for the modeled A2 (Figure S4). This resulted in an overall displacement of the Cα backbone of A2 in the crystal structure. As a result, E304 was also positioned too far away from binding to R101 and K102.

Therefore, the individual amino acid contributions for A1–A4 to the total contact energies were very different from the modeled versus the crystal structures (Table 1 vs. Table 2). Importantly, the three identified ‘hot spots,’ namely positions 297, 298, and 304 of the natural TAF8 NLS, lost their novel contacts formed in the modeled structures. These changes resulted in overall less contact energies being formed for mImpα2 in the crystal structures compared to the energies from the modeled analogs for human importin‐α1 (Table 1 vs. Table 2). Nevertheless, the binding modes were highly similar and A1, A3, and A4 had cumulative contact energies to mImpα2 comparable to the contact energy that TAF8 NLS had with human importin‐α1 (Table 1). In addition, it cannot be excluded that these deviations in binding energies were driven by crystallization conditions (and that in solution these binding energies will be similar to those calculated in the original in silico model).

### 2.5 Binding of the zero net‐charge analogs at the minor groove

**FIGURE 4:**
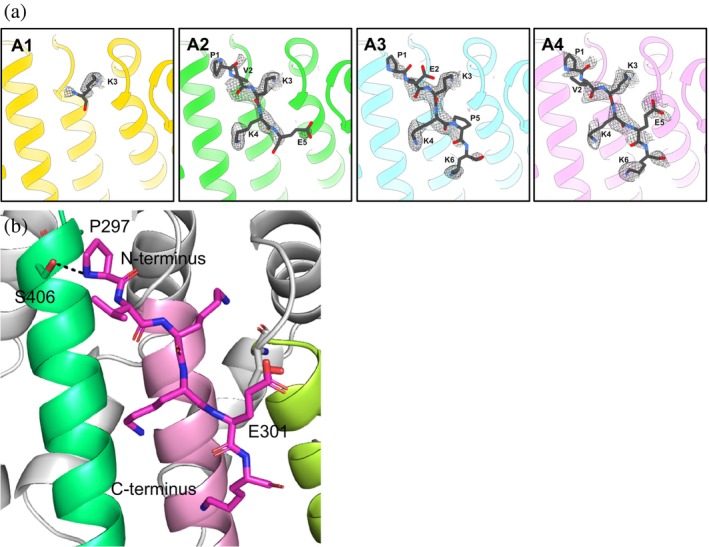
Resolved crystal structures of the zero net‐charge analogs at the minor groove of mImpα2. (a) The Fo–Fc difference maps of A1–A4 at the major groove of mImpα2. The A2–A4 analogs in the minor NLS‐binding groove have increased prominence and rigidity compared to TAF8 NLS (Trowitzsch et al. 2015). (b) Notable differences in the binding modes between the analogs (A4 shown) in the minor groove as opposed to the major groove (Figure 4) of mImpα2. ARMs 6–8 colored lime, pink, and lime green, respectively.

In the original TAF8 NLS–human importin‐α1 complex, the electron density in the minor groove was insufficient to resolve the NLS, and because the ITC measurement determined a *K*_D_ value on the low end of the affinity spectrum for monopartite NLSs, it was concluded to be an exclusively major groove‐specific binder (Trowitzsch et al. 2015). By contrast, residues 297–302 for A3 and A4 were clearly defined in the minor groove (Figure 4a). A3 had the best cumulative interaction energy (−43.46 kcal/mol) at the minor groove and formed 9 hydrogen bonds, 2 salt bridges, 27 VdW interactions, and 1 cation–π bond. A3 buried 504.2 Å^2^ of surface area.

The P′0 site (counterpart of P1 of the major groove) of the NLS‐binding minor groove is defined by a pocket with two sharp peaks formed by T409 and W399. In contrast to the hairpin turn motif made by N‐terminal proline residue‐containing NLSs at the major groove, P297 for both A3 and A4 was straight and positioned near S406 and formed hydrogen bonds (Figure 4b). As anticipated, K299 bound at the critical P′1 site (counterpart of P2). However, the binding energies are dominated by K300 and K301 at positions P′2 and P′4. E298 and E301 from A3 and A4, respectively, were the only acidic amino acids introduced that were resolved at the minor groove, and they formed multiple VdW interactions. The core NLS consensus motif dominated the interaction energies, accounting for nearly 100% and 91% of the total binding energy for A3 and A4, respectively.

### 2.6 Zero net‐charge analogs demonstrate effective binding to mImpα2 and drive localization to the cell nucleus, potentially favoring the minor groove

In the presence of TAF8 NLS, mImpα2 was stabilized, evidenced by the increase in temperature denaturation, shift in stability curves, and the reduction of the unfolded state (Figures S5a–c and 5a). All thermodynamic results are listed in Table S1. The analogs also stabilized mImpα2, albeit to a lesser extent than TAF8 NLS. The stabilization order was A3 > A1 > A4 ≈ A2.

**FIGURE 5:**
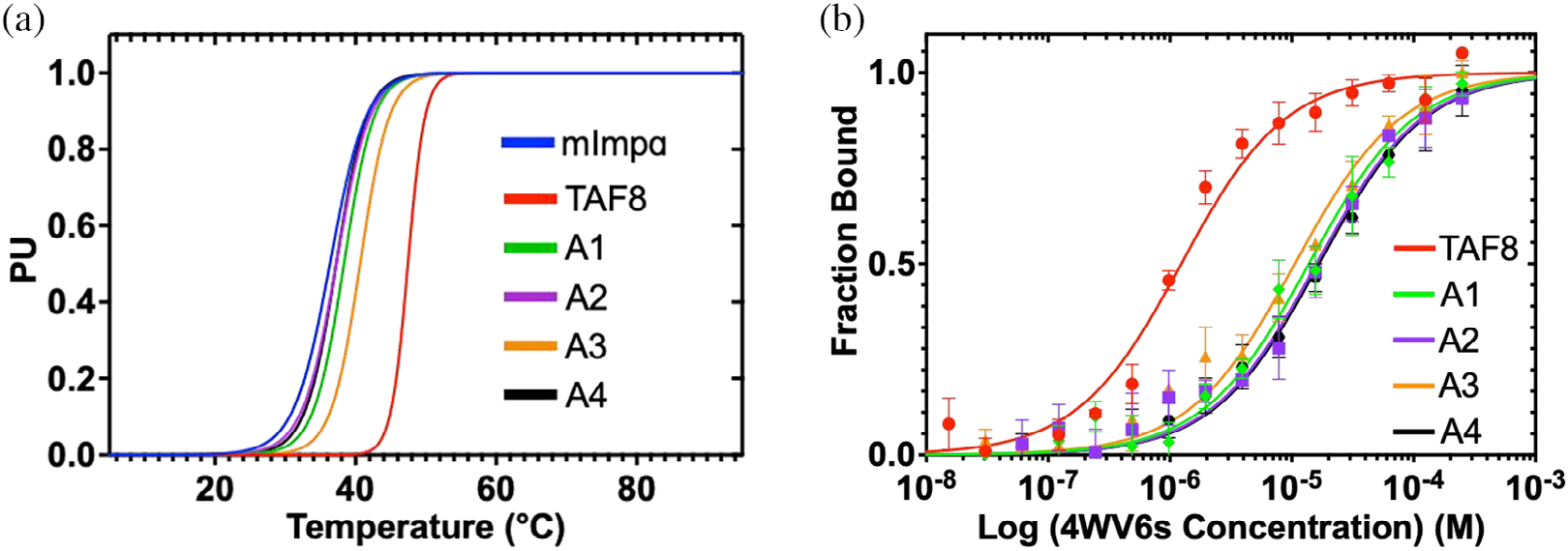
Circular dichroism (CD) and microscale thermophoresis (MST). (a) The proportion of the unfolded state as a function of temperature. Data was fitted from the thermal denaturation profile (Figure S5b). (b) The logarithmic scale binding affinities using a one‐binding site model.

Fitting the MST data to a one‐binding site model (Figures S5d and 5b), TAF8 NLS exhibited the highest apparent affinity, with *K*_D_ values approximately 10‐ to 16‐fold higher than those of the analogs (Table S2). Trowitzsch et al. originally reported *K*_D_ value for TAF8 NLS of ~10 μM with 1:1 stoichiometry (Trowitzsch et al. 2015). Due to our crystallography findings revealing the presence of the analogs at both binding grooves, in contrast to TAF8 NLS strictly in the minor groove, the TAF8 NLS and the zero net‐charge analog binding isotherms were also fitted with a two‐binding site model. In contrast to Trowitzsch et al., the binding affinities were both ~1 μM for both sites and suggest TAF8 NLS actually binds at both grooves.

Here, the *K*_D_ values for the analogs showed significant disparity between the two sites (Table S2). The *K*_D_ values at site 1 (*K*_D_1) ranged from 3‐ to 6‐fold weaker, while the *K*_D_ values at site 2 (K_D_2) were approximately 28‐fold weaker than TAF8 NLS. To graphically discern two binding phases to two different binding sites, the two combined *K*_D_ values of 1 μM for *K*_D_1 and *K*_D_2 amounted to 100% of bound mImpα2 (Figure S6). Hill coefficient analyses supported the fitted data (Tso et al. 2018), indicating that TAF8 NLS binds mImpα2 with positive cooperativity, while the analogs bind with lower cooperativity (Table S2 and Figure S7).

**FIGURE 6:**
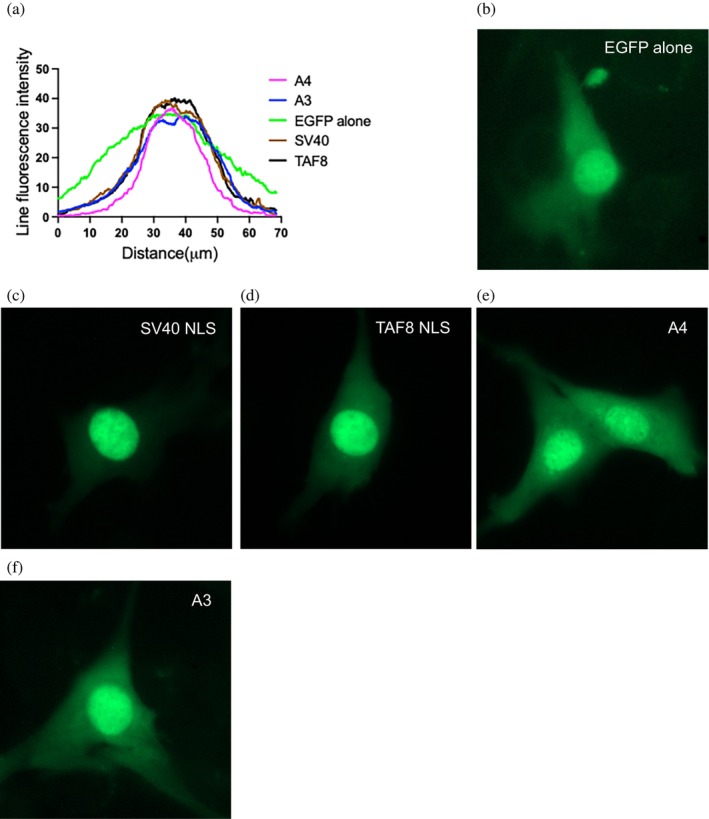
Fluorescence microscopy. (a) The overlaid line fluorescence intensities for C2C12 myoblasts transfected with the different pCIneoEGFP plasmids. (b–f) Representative C2C12 myoblasts fluorescence microscopy images transfected with different constructs.

To test the functionality of the zero net‐charge approach in generating novel NLSs, mammalian cell lines were transfected with constructs encoding enhanced green fluorescent protein (EGFP) fused to canonical NLSs (SV40 and TAF8), or the TAF8‐derived A3 and A4 analogs. Nuclear localization was assessed by fluorescence microscopy (Figure 6).

Line overlay analysis of fluorescence intensity across C2C12 myoblasts revealed sharp increases in fluorescent nuclear accumulation for EGFP‐A3 and EGFP‐A4, with peak fluorescence localized within the ~20–40 μm zone corresponding to nuclear regions (Figure 6a). This nuclear accumulation was comparable to that observed in cells expressing EGFP‐TAF8 NLS and EGFP‐SV40 NLS fusion proteins. In contrast, myoblasts expressing EGFP alone exhibited only a shallow increase within the nuclear region, indicative of passive diffusion.

Peak fluorescence intensity was highest in cells transfected with EGFP‐TAF8 NLS and EGFP‐A3, both of which displayed strong nuclear‐specific fluorescence and sharp contrast relative to the cytoplasm (Figure 6d,f). EGFP‐SV40 NLS showed robust and crisp nuclear‐specific fluorescence intensity, consistent with the known strength of this canonical NLS. Conversely, EGFP alone remained diffusely distributed throughout the cell (Figure 6b), further supporting its lack of nuclear targeting and passive diffusion. Fluorescence imaging also indicated active nuclear localization of EGFP‐A4 but to a lesser extent than EGFP‐A3 (Figure 6e).

Experiments were also performed to determine whether nuclear localization was energy‐dependent, where the RanGDP/GTP gradient is deactivated (details in Data S1) and previously shown to inhibit active nuclear import (Schwoebel et al. 2002). However, no differences in nuclear localization were observed across constructs or at multiple time points post‐transfection to the results obtained with standard transfection and imaging, as shown in Figure 6.

Similar trends were observed in Hela cells, where EGFP‐NLS constructs exhibited sharply elevated nuclear fluorescence relative to EGFP alone (Figure S8a–f). Interestingly, the total nuclear localization intensities were less than half observed in C2C12 myoblasts across constructs. In this context, A4 outperformed A3 in promoting nuclear localization. Additional wide‐field images, including DAPI staining and representative fields of broader cell populations, are shown in Figure S8g,h. Taken together, these findings demonstrate that the TAF8‐derived zero net‐charge analogs, A3 and A4, are functional NLSs in the cells evaluated in this study.

**FIGURE 7:**
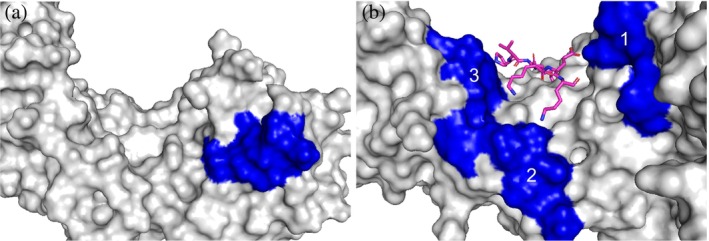
The cationic surface of the major and minor NLS‐binding groove of mImpα2. Surface renderings of the (a) major and (b) minor NLS‐binding grooves of mImpα2. The blue regions are the cationic surfaces. Cationic patches 1–3 are labeled in the minor site. The resolved crystal structure of A4 (pink) in the minor site is shown. The major groove cationic patch is composed of residues G72, T73, V74, Q98, R101, K102, and W142 and has a surface area of 70 Å^2^. The minor site has three cationic patches. Patch 1 consists of R238, N239, K240, N241, and Y277. Patch 2 consists of K348, T349, N350, and K353. Patch 3 consists of F391, K392, K395, W399, K435, Q438, and V439. Cationic surfaces were determined using MOE and images rendered in Pymol.

Analysis of the minor and major NLS‐binding grooves revealed very interesting aspects regarding their respective cationic surfaces. The minor groove, with three cationic patches, has a combined surface area 342% larger than the major groove (Figure 7). The three cationic patches are all grouped at the N‐terminus that contains positions P′4 and extend to the edges of the binding groove and suggest they can engage with the acidic amino acids at the C‐termini of the NLS analogs. This is supported by analogs A3 and A4 having most of their residues resolved at the minor groove (Figure 5). This also supports why TAF8 NLS was not resolved at the minor groove, as its C‐terminal arginine residues could have strongly been repulsed by the cationic surfaces.

Although specific *K*_D_ values cannot be assigned to either the major or the minor NLS‐binding grooves, these findings demonstrate that the zero net‐charge analogs effectively bind to and stabilize mImpα2, with affinities indicating a preference for the minor groove. The crystal structures suggest that while TAF8 NLS binds exclusively to the major groove, the consistent *K*_D_ values suggest that it too can interact with both sites; this is sufficient to target the nucleus in the cell lines evaluated in this study.

## 3 DISCUSSION

In this study, novel NLS tags with zero net‐charge were developed with the aim to potentially improve the pharmacokinetics and reduce the non‐specific interactions associated with traditional NLS‐tagged therapeutics (Beaudoin et al. 2016; Chen et al. 2006). Four zero net‐charge NLS analogs were successfully designed using structure‐guided computational modeling, achieving docking scores and binding modes at the major site of importin‐α comparable to TAF8 NLS. Crystal structures of these analogs complexed with mImpα2 confirmed their binding modes, albeit the novel interactions formed in the modeling procedure were disrupted by DTT used during crystallization. CD and MST experiments revealed the analogs bound mImpα2, albeit with reduced *K*_D_ values. Among these, analogs A3 and A4 demonstrated functional NLS activity in both C2C12 myoblasts and Hela cells and favored the minor groove. These results reveal the feasibility of engineering noncanonical zero net‐charge NLSs that retain importin‐α binding capacity and nuclear import function.

The positively charged amino acids that interact at the P2, P3, and P5 sites, whose side chains are deeply buried in negatively charged cavities in the major binding groove, are critical for TAF8 NLS binding. The binding of these NLS amino acids at these P sites of the major groove of importin‐α is foundational in classical nuclear transport. Currently, TAF8 NLS is considered a major groove‐specific binder, even though its canonical consensus sequence would also suggest binding at the minor groove. One can argue TAF8 NLS was too mobile to be modeled entirely at the minor groove in the original study (Trowitzsch et al. 2015). In support of this perspective, the N‐terminus of TAF8 NLS dominates the crystal packing interactions with the C‐terminal arginine residues notably located outside the pocket and stabilized by water molecules with no interactions to importin‐α (Figure 1a,b). TAF8 NLS would appear to bind even less at the minor groove considering C‐terminal arginine residues would interact right at the area of extensive cationic surfaces (Figure 7).

However, our MST data provide evidence that TAF8 NLS binds both sites with affinities of 1 μM, which is 10‐fold stronger than previously reported (Trowitzsch et al. 2015). This discrepancy may be due to methodological differences, where the previous study used ITC and where the binding of two sites with equivalent affinity could have been interpreted as binding to only one site.

The following question naturally arises—How can we reconcile the fact that more total (major + minor grooves) electron density translated into lower apparent binding affinities for the zero net‐charge analogs? Fitting a two‐binding site model resulted in *K*_D_ values ranging from *K*_D_1 = 5 to 7 μM and *K*_D_2 = 30 to 40 μM. Although it was not possible to assign which *K*_D_ value belongs to which binding site, due to general electrostatics principles, the highly positively charged TAF8 NLS may have a much higher association rate constant and/or lower dissociation rate constant (*k*_off_) than the analogs at the major groove. But at the minor groove, our results suggest that the analogs with acidic amino acids at their C‐termini can bind better at the minor groove due to the extensive cationic surface, which provides more interaction opportunities, despite these residues not being resolved in the crystal structures and might provide them with at least lower *k*_off_ values. Therefore, this suggests that the *K*_D_1 values reflect binding of the analogs at the minor groove (Table S2).

Nuclear accumulation of A3 and A4 in cell‐based assays supports their classification as active NLSs (Figures 6 and S8). These analogs, when fused to EGFP, localized sharply to the nucleus in both myeloblasts and Hela cell contexts. It is possible that a portion of the nuclear accumulation exhibited by the EGFP‐NLS constructs was due to passive diffusion due to the molecular weight of EGFP being under the threshold of the NPC (Timney et al. 2006). Nevertheless, the clear differential localization relative to EGFP alone further confirms importin‐α‐mediated import.

From a therapeutic perspective, it is important to note that there are typically 10–20 NLSs per therapeutic antibodies (Beaudoin et al. 2016; Chen et al. 2006), which allows multiple NLS sampling at the minor and major grooves, and the potential cumulative cooperativity effect would enable major and minor groove sampling redundancy and further enable effective binding and nuclear localization. It has been demonstrated that low‐affinity molecules targeting minor pockets of importin‐α can exert antiviral activity, where synthesized minor‐specific small‐molecule compounds exhibiting millimolar *K*_D_ blocked virus nuclear localization (Nakada et al. 2015). The zero net‐charge analogs displayed low micromolar affinities to, most likely, the minor groove and, thus, are very promising for the development of NLS therapeutics.

However, the relationship between affinity and nuclear import remains complex. The field of nuclear import faces a critical challenge in harmonizing NLS affinity measurements and their impact on *K*_D_ values with nuclear import efficiency. Small differences (3‐fold) in NLS/importin‐α affinities have been shown to significantly affect the formation of transport complexes (Timney et al. 2006; Wirthmueller et al. 2013). Conversely, it has also been shown that efficient nuclear import can tolerate NLS mutations diminishing importin‐α binding affinity by up to 200‐fold (Alvisi and Jans 2014; Harreman et al. 2004; Hodel et al. 2001). The SV40 NLS, which is a foundational NLS for understanding binding principles (Fontes et al. 2000) and widely utilized in NLS‐therapeutic design (Goswami et al. 2024), has a perplexing disparity (1500‐fold difference) in the reported *K*_D_ values (10 nM–15 μM) measured by ELISA, ITC, fluorescence depolarization, and surface plasmon resonance (Catimel et al. 2001; Fanara et al. 2000; Fontes et al. 2003; Sankhala et al. 2016). In this study, the isotherm fits were of good statistical quality as the MST data had low standards of deviations between replicates. Additionally, MST was able to process multiple samples near‐concurrently in a short time.

Another important consideration is the role of the IBB. The IBB domain exhibits micromolar affinity for importin‐α (*K*_D_ ≈ 14 ± 5 μM), which varies between importin‐α members and nanomolar affinity for importin‐β (*K*_D_ ≈ 10 nM) (Harreman et al. 2003). The presence of importin‐β enhances binding affinity for NLS by ~30‐fold (Harreman et al. 2003).

A growing body of work is linking NLS charge with biological outcomes. Although it is currently unknown how natural eukaryotic NLS‐containing proteins determine their rates and levels of nuclear localization with respect to charge, there is evidence from viral pathogenicity. For example, the NLS charge is associated with coronaviruses pathogenicity. Coronaviruses with highly cationic classical NLSs on their nucleocapsid proteins, including human SARS Coronavirus 2, ranked highest in pathogenicity and transmission compared to coronaviruses with NLSs with less cationic charge (Gussow et al. 2020).

In conclusion, this study analyzed the structural and energetic landscape of the NLS of human TAF8, shedding deeper insight on its binding to importin‐α. We used these insights to design zero net‐charge analogs and showed their ability to effectively target mImpα2 with a preference for the minor groove. These findings support a revised model of NLS design that emphasizes groove selectivity, charge modulation, and tunable affinity. Future studies will explore integrating zero net‐charge NLSs into antibody‐based therapeutic platforms.

## 4 MATERIALS AND METHODS

### 4.1 In silico TAF8 NLS‐based analog design and docking

The Protein Data Bank (PDB) file 4WV6 for the TAF8 NLS complexed with human importin‐α1 was loaded into Molecular Operating Environment (MOE; Chemical Computing Group, Montreal, Canada). Individual residues underwent alanine substitution followed by docking to generate 100 docking poses and associated dAffinity scores (kcal/mol). The distribution of differences in dAffinity scores between the natural NLS and the derivatives containing alanine substitutions at different positions was determined. If the distributions followed the normal law, the Welch *t* test was utilized to determine if there was significance (*p* ≤ 0.05) between the means. In non‐normality scenarios, the Mann–Whitney *U* and Hodges‐Lehmann tests were also utilized (Lumley et al. 2002; Vaux 2014). As a result, only amino acids that were not sensitive during alanine scanning were pursued for substitution with different amino acids, with the goal of generating sequences with net zero charge. Residue scanning was then performed on the non‐sensitive amino acids for the given NLSs, and docking and statistical analysis were performed as previously described.

It was crucial for zero net‐charge analogs to match the binding mode of the natural TAF8 NLS. Therefore, prior to testing different zero net‐charge analogs, the docking procedure had to ensure that it would not cause non‐natural binding modes. Docking of the in silico designed natural TAF8 NLS was performed. The Cα's of the in silico TAF8 NLS superposed very well to its NLS counterpart crystal structure. The NLS analogs were then docked and the best candidates with comparable docking scores to TAF8 NLS were selected for advancement.

### 4.2 Analog synthesis

TAF8 NLS and analogs were synthesized at the Peptide and Imaging Probe Synthesis Platform (Université de Sherbrooke) based on a similar method (Beaudoin et al. 2016); further details are described in Data S1 and their characteristics are reported in Table S3.

### 4.3 DNA constructs, protein production and purification

For biophysical investigations, the pET30a vector plasmid containing mImpα2 cDNA, lacking the importin‐β‐binding domain, and featuring the E396R mutation, was kindly provided by Professor Bostjan Kobe (The University of Queensland, Australia). Site‐directed mutagenesis was applied to revert the deletion to its native sequence. Transformation, expression, and purification details are described in Data S1.

### 4.4 Crystallization and structure determination

MImpα2, residues 70–529, was co‐crystallized with each NLS analog using the sitting drop vapor diffusion method at 20°C. Briefly, 1 μL of protein (18.3 mg/mL) was mixed with 1 μL of reservoir solution and 0.7 μL of each peptide (3 mg/mL) and suspended over 0.5 mL of reservoir solution, resulting in a molar ratio of 5.2:1 for each analog over mImpα2 protein (Fontes et al. 2000). Crystals were grown for 4 weeks and were harvested with the addition of 1 μL cryoprotectant (0.6M sodium citrate, 100 mM HEPES pH 6.4 and 20% glycerol) and flash‐frozen in liquid nitrogen. Detailed crystallization conditions for each crystal are listed in Table 3 and details on the collection of diffraction datasets and structure determination can be found in Data S1.

### 4.5 Apparent NLS–mImpα2 complex stability monitored by CD

CD measurements were conducted as previously described, including the determination of free energy using the Van't Hoff method (Jean‐Francois et al. 2003). Fitting details are in Data S1.

### 4.6 Microscale thermophoresis

Microscale thermophoresis (MST) experiments were conducted using the Monolith NT.115 (Nanotemper, Germany). The RED‐NHS 2nd generation Nanotemper labeling kit (MO‐L011) was used to label mImpα2 in 20 mM Tris–HCl and 127 mM NaCl at pH 8.0. Ten microliter of RED‐NHS‐reacted mImpα2 at 60 nM was titrated against a serial 1:1 dilution (250 μM–7.6 nM) of 10 μL of NLS containing 0.5% Tween‐20, and the mixture was pipetted into 16 MST capillaries contained in a single cartridge. The samples were processed using default settings and followed the manufacturer's protocol. The binding model fits are detailed in Data S1.

### 4.7 Fluorescence microscopy

The plasmids, cloning, and cell culture transfection methods are described in Data S1. Cells were fixed with 3% paraformaldehyde (Electron Microscopy Sciences, Cat#15710) for 15 min at room temperature; then rinsed thoroughly with PBS. Nuclear staining was performed using 1 μg/mL DAPI (Sigma, Cat#D9542) in PBS. Following staining, cells were rinsed again with PBS and mounted with glass coverslips using Vectashield Antifade Mounting Medium (Vector Laboratories, Cat#H‐1000).

Fluorescence imaging was performed using a Zeiss AxioImager.M2 microscope (RRID:SCR_024706) equipped with a 40× EC Plan‐NeoFluar (1.3 NA; oil) objective, X‐Cite XYLIS LED illumination system, and a Zeiss AxioCam mRm CCD monochrome camera. Image acquisition and initial processing were performed using ZEN Blue software (RRID:SCR_013672), and additional analyses were performed using Fiji (ImageJ; RRID:SCR_002285). For each experimental condition, 15 randomly selected fields of view were imaged. Line overlay fluorescence intensity was quantified using the line profile tool in Fiji. A straight line across the entire cell length was drawn and centered on the nucleus. The “Plot Profile” function was used to extract pixel intensity values along the line. Three cells were analyzed per condition, and the data were presented as mean fluorescence intensity values.

### 4.8 Coordinates

Structures and maps have been deposited in the PDB (9CL8, 9CLE, 9CLF, and 9CLG).

## AUTHOR CONTRIBUTIONS

**Amirabbas Abdoli:** Investigation; methodology; writing – review and editing; formal analysis. **Zhihan Yang:** Investigation; methodology; writing – review and editing; formal analysis. **Abdullah Odeh‐Ahmed:** Methodology. **Olga Bednova:** Methodology. **Bruno Lemieux:** Methodology. **Leanne Dawe:** Methodology; visualization. **Aymeric Ravel‐Chapuis:** Methodology; writing – review and editing; visualization; formal analysis. **Pierre Lavigne:** Conceptualization; writing – review and editing; supervision; formal analysis; resources. **Natalie Zeytuni:** Conceptualization; funding acquisition; writing – review and editing; formal analysis; supervision; resources; visualization; software. **Jeffrey V. Leyton:** Conceptualization; funding acquisition; writing – original draft; visualization; writing – review and editing; formal analysis; project administration; supervision; software.

## Supporting information


**Data S1.** Supporting Information.

## Data Availability

The data that support the findings of this study are available on request from the corresponding author. The data are not publicly available due to privacy or ethical restrictions.
