# Supplementary material for: Probing the extent of importin‐α targeting of the TAF8 NLS by eliminating its cationic net‐charge
Source: Protein Sci. 2025 Aug 25;34(9):e70272. doi: 10.1002/pro.70272 (PMC12375970; doi:10.1002/pro.70272)
Supplement: Supplementary file 1 — Data S1. Supporting Information. [file PRO-34-e70272-s001.docx]

**SUPPLEMENTAL RESULTS**

**Supplemental results, figures, and tables**

**Modeling of TAF8, SV40 and c-Myc NLSs onto murine importin-ɑ isoform 2 (mImpɑ2) and Supplemental Figure 1.**

To further investigate the potential of substituting amino acids at the N-terminal region of TAF8 NLS, we modeled it, SV40 and c-Myc NLSs onto mImpɑ2, for four main reasons. One, mImpɑ2 is the most studied importin-ɑ for evaluating structural interactions with NLSs and used for the crystallography results described in a subsequent result section. Two, mImpɑ2 is very similar to human importin-ɑ1 (used for TAF8) with a sequence similarity of 98% and 100% within the NLS-binding grooves and has an RMSD fit of 0.69 Å [1]. Three, SV40 and c-Myc both have N-terminal proline residues that slot at the P1 site and contain a hairpin-turn [2, 3]. Lastly, each resolved structure of these NLS are bound to importin-ɑ from different species (TAF8:human, SV40:mouse, and c-Myc:yeast), and it will be more accurate to understand their binding modes in the NLS-binding groove of a single importin-ɑ.

Through our modeling approach, a high degree of alignment among the NLSs, particularly at the most important P2 site of the major NLS-binding groove of mImpɑ2. Overall, there were no drastic changes in the binding modes of the three NLSs. The interactions and energies with amino acids critical for P2 site-binding with the prime NLS lysine residue, (K128 for SV40, K299 for TAF8, K323 for c-Myc) were very close as evidenced by their cumulative interaction energies of -31.01 kcal/mol, -30.09 kcal/mol, and -30.14 kcal/mol, respectively. For all three NLSs, the tightest interaction with mImpɑ2 is mediated by hydrogen bonds with side chain D192 at the P2 site. In the context of the core NLS motif, the strongest contacts made for all three NLSs were by their amino acids that slotted into the P2, P3, and P5 sites, and strongly support the consensus sequence for NLSs utilized in classical nuclear transport. The SV40 NLS had the overall tightest binding (-116.67 kcal/mol) followed by TAF8 (-104.05 kcal/mol) and c-Myc (-101.49 kcal/mol).

Our analysis revealed common spatial orientations and interactions of the N-terminal proline residues at the P1 site of the major NLS-binding groove of mImpɑ2 (Suppl. Fig. 1). P297 and V298 of the TAF8 NLS maintained the same contacts as in the crystal structure where they bound to human importin-ɑ1 (Fig. 1C), defined by their C-terminal carboxyl groups forming hydrogen bonds with the side chains of W231 and N235 (Suppl. Fig. 1A). The c-Myc NLS contains two amino acids as opposed to one residue like TAF8 and SV40 between the P1 and P2 sites. However, the N-terminal P320 sits up and out of the P1 site like the proline residue counterparts for the TAF8 and SV40 NLSs. Its position is stabilized by its C-terminal carboxyl group forming a hydrogen bond with the side chain of R238 (Suppl. Fig. 1B). Additionally, the C-terminal carboxyl group of A322 forms a hydrogen bond with N235. The pyrrolidine side chain of P126 of the SV40 NLS orients itself very similar to the proline counterpart of the TAF8 NLS (Suppl. Fig. 1C). The C-terminal carboxyl groups of P126 and K127 form identical hydrogen bonds with the side chains of W231 and N235. The carboxyl group of K127 forms an additional, but weakly energetic, hydrogen bond with G191 in ARM3.

This modeling analysis of the contacts of the N-terminal proline region with mImpɑ2 showed that the binding energies for this unique ‘proline motif’ stem exclusively from the C-terminal carboxyl groups of this amino acid (Suppl. Fig 1.). Additionally, the pyrrolidine side chains of the proline residues are positioned above the binding groove as opposed to buried within the groove basin. Although adjacent non-polar amino acids seem critical, they do not appear to form any meaningful contacts with mImpɑ2, compared to the essential positive charged residues that bind the P2, P3, and P5 sites. Therefore, both the N-terminal P297 and V298 are candidate positions for substitution for the development of zero net-charge NLSs.

**Supplemental Figure 2.**


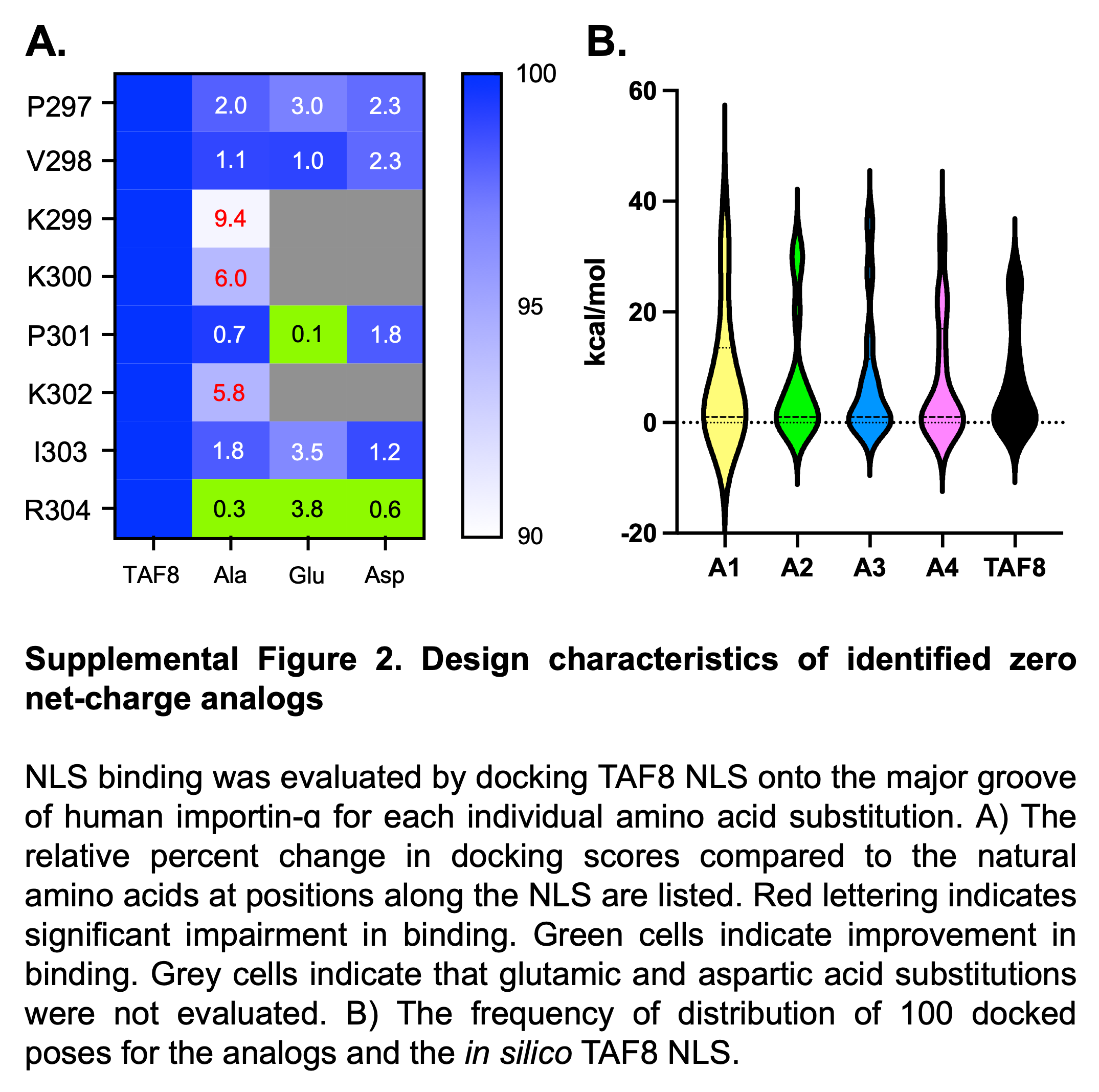


**Supplemental Figure 3.**

**
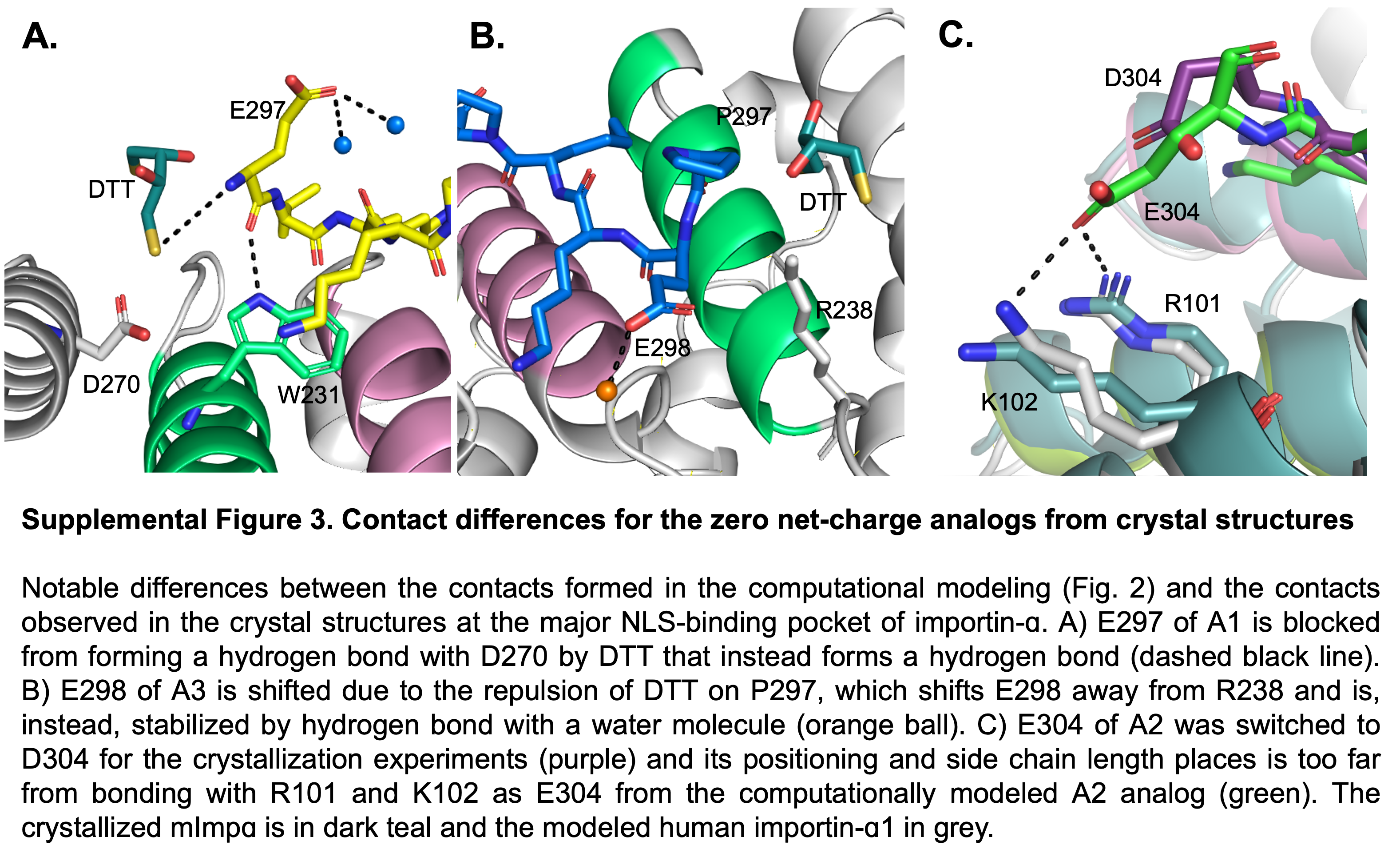
**

**Supplemental Figure 4.**

**
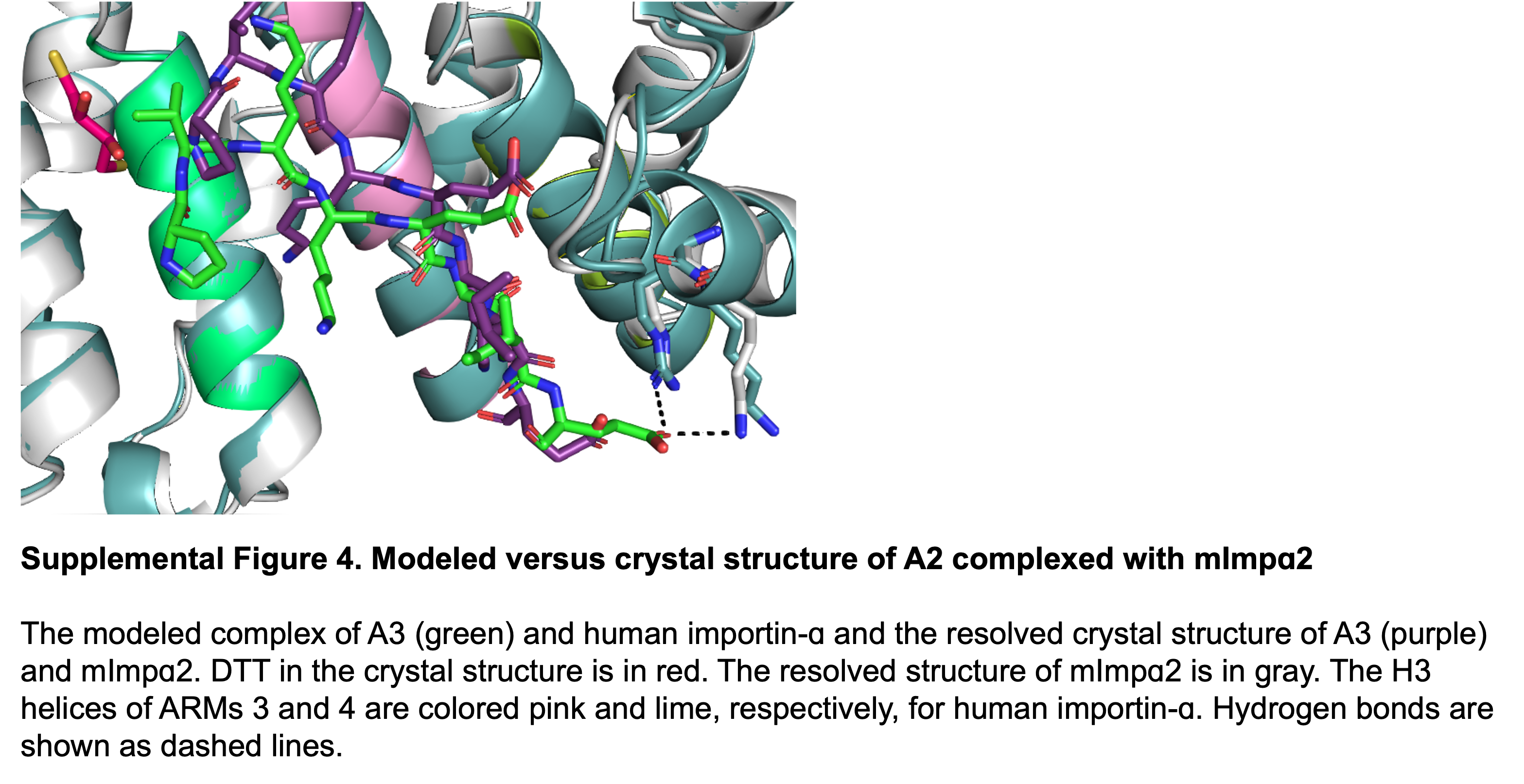
**

**Supplemental Figure 5.**

**
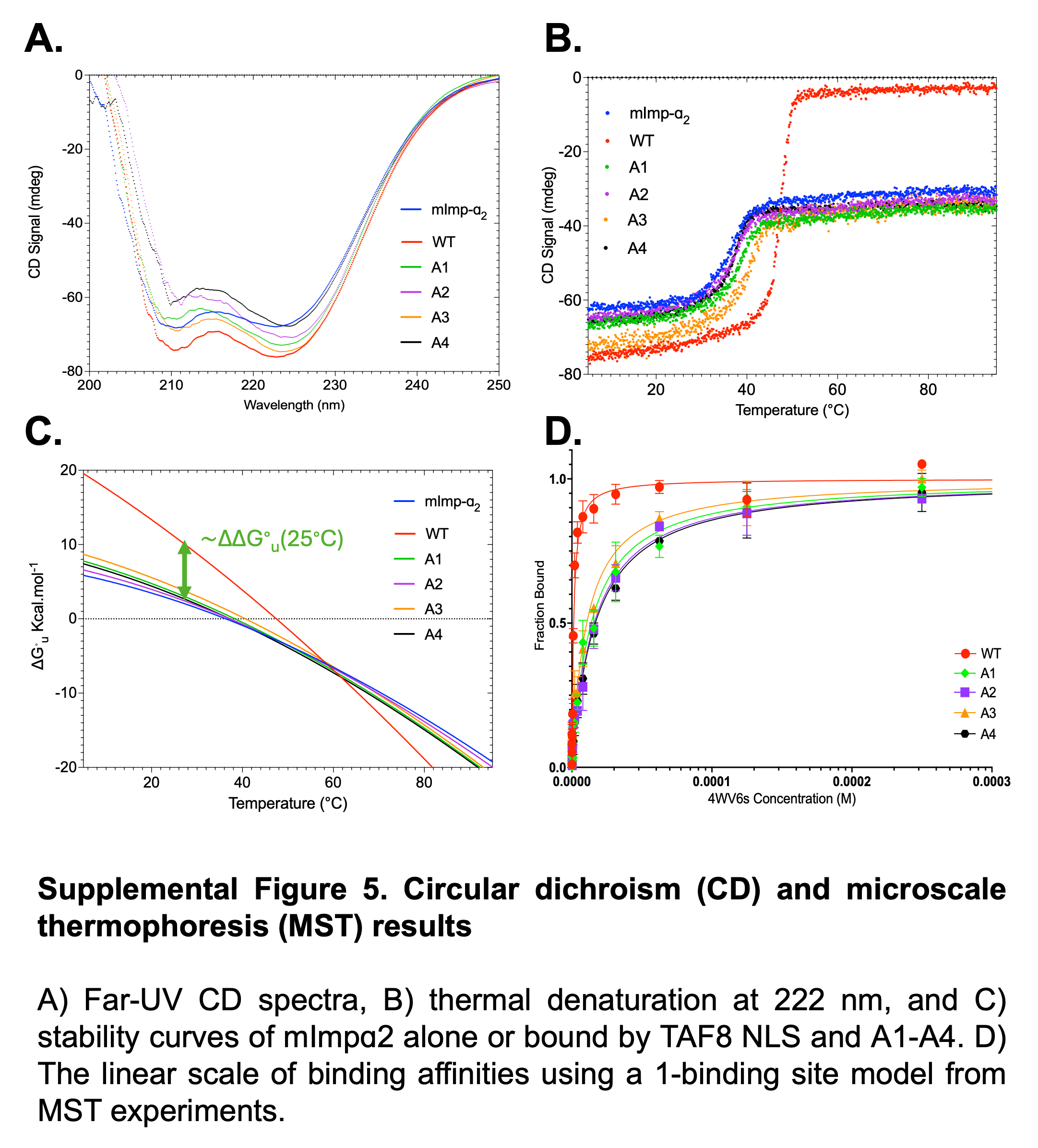
**

**Supplemental Table 1**: **Thermodynamic results**

| Ligands | T° (°C) | ∆H°_u_ (T°)^a^ | ΔG°_u_ (25°C)^a^ | ΔΔG°_u_ (25°C)^a^ | ΔG°_u_ (37°C)^a^ | ΔΔG°_u_ (37°C)^a^ |
| --- | --- | --- | --- | --- | --- | --- |
| mImp⍺_2_ | 36.35 | 74 | 2.5 | 0 | -0.2 | 0 |
| TAF8 | 47.45 | 170 | 11.1 | 8.6 | 5.4 | 5.6 |
| A1 | 38.35 | 90 | 3.6 | 1.1 | 0.4 | 0.6 |
| A2 | 37.25 | 80 | 2.9 | 0.4 | 0.1 | 0.3 |
| A3 | 40.55 | 94.9 | 4.3 | 1.8 | 1.1 | 1.3 |
| A4 | 37.15 | 87.9 | 3.2 | 0.7 | 0.1 | 0.3 |

^a^Values in kcal/mol

| Supplemental Table 2. K_D_ values from MST data | | | | | |
| --- | --- | --- | --- | --- | --- |
|  | **1-binding site fitted model** | **2-binding site fitted model**  **Site 1 Site 2** | | **Hill coefficient model** | |
| Ligand | K_D_ (µM) | K_D_1 (µM) | K_D_2 (µM) | K_D_ (µM) | Hill Coeff. |
| TAF8 | 1.21 ± 0.313 | 1.19 ± 0.37 | 1.22 ± 0.22 | 1.54 ± 0.05 | 3.1 ± 0.39 |
| A1 | 13.76 ± 2.31 | 5.66 ± 0.99 | 33.47 ± 5.65 | 17.48 ± 5.01 | 0.8 ± 0.34 |
| A2 | 15.88 ± 3.1 | 7.2 ± 1.73 | 34.55 ± 7.21 | 20.96 ± 8.77 | 0.73 ± 0.32 |
| A3 | 10.55 ± 2.14 | 3.58 ± 0.74 | 29.66 ± 5.32 | 16.01 ± 7.16 | 0.71 ± 0.30 |
| A4 | 16.65 ± 1.96 | 6.7 ± 1.0 | 40.77 ± 5.58 | 24.24 ± 4.6 | 0.64 ± 0.24 |

**Supplemental Figure 6.**


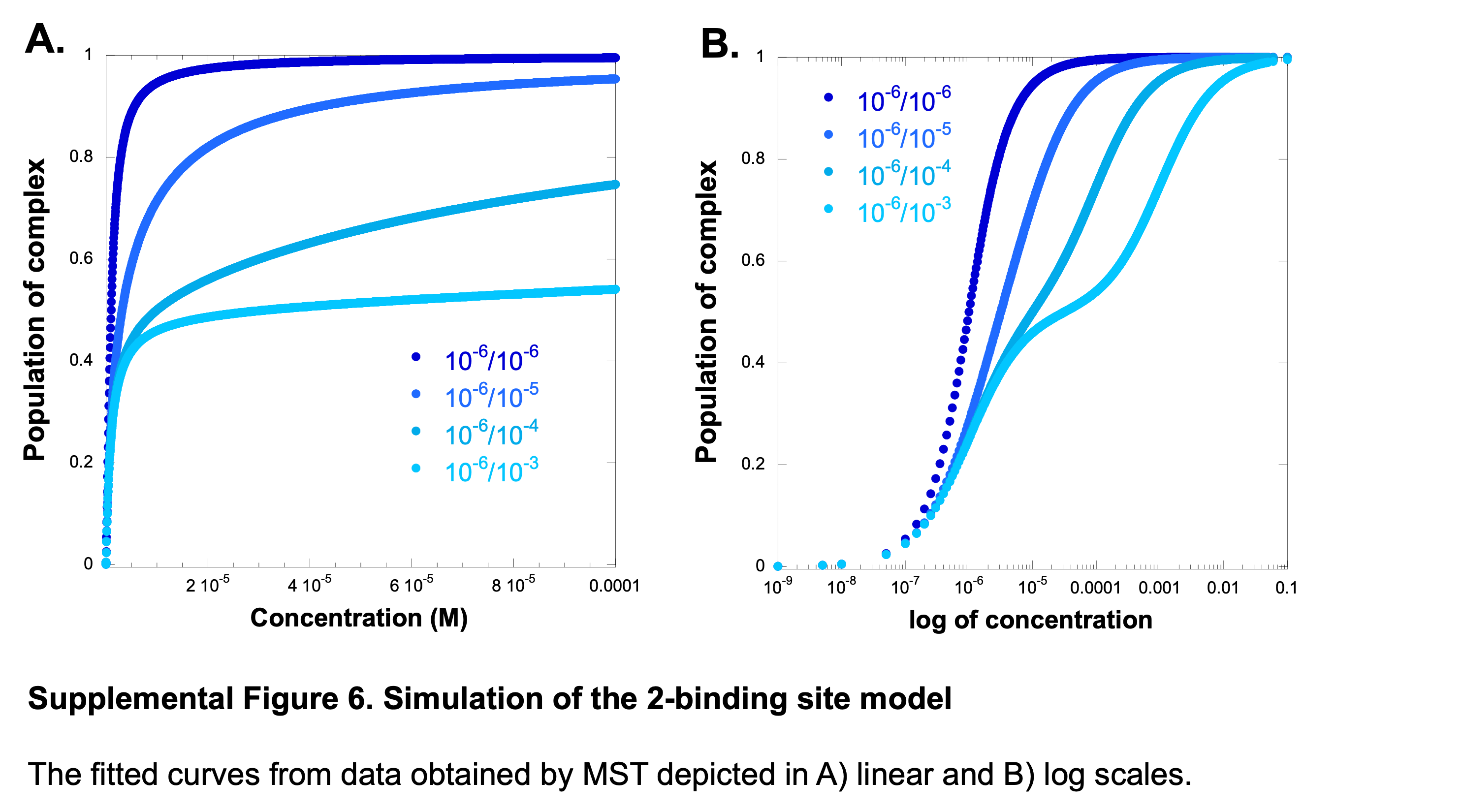


**Supplemental Figure 7.**

**
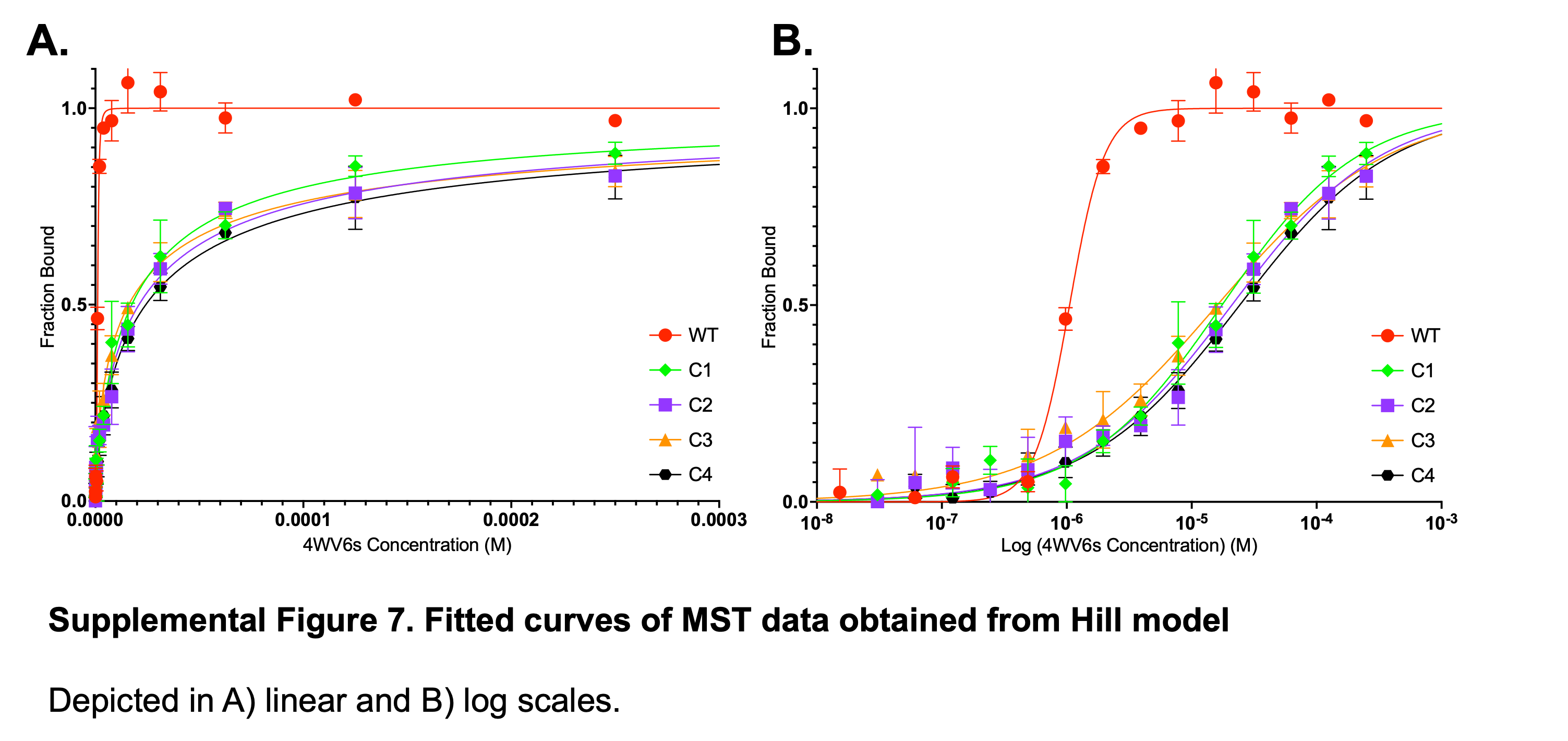
**

**Supplemental Table 3**: TAF8 NLS analog characteristics

| NLS | Mass (Da) | Purity | Quantity (mg) | Soluble in H_2_0 |
| --- | --- | --- | --- | --- |
| TAF8 | 1,121.42 | >99% | 33.9 | Yes |
| A1 | 1,085.25 | >99% | 20 | Yes |
| A2 | 1,099.27 | >99% | 14.7 | Yes |
| A3 | 1,083.23 | >99% | 32.6 | Yes |
| A4 | 1,085.25 | >99% | 18.2 | Yes |

**Supplemental Figure 8.**

**
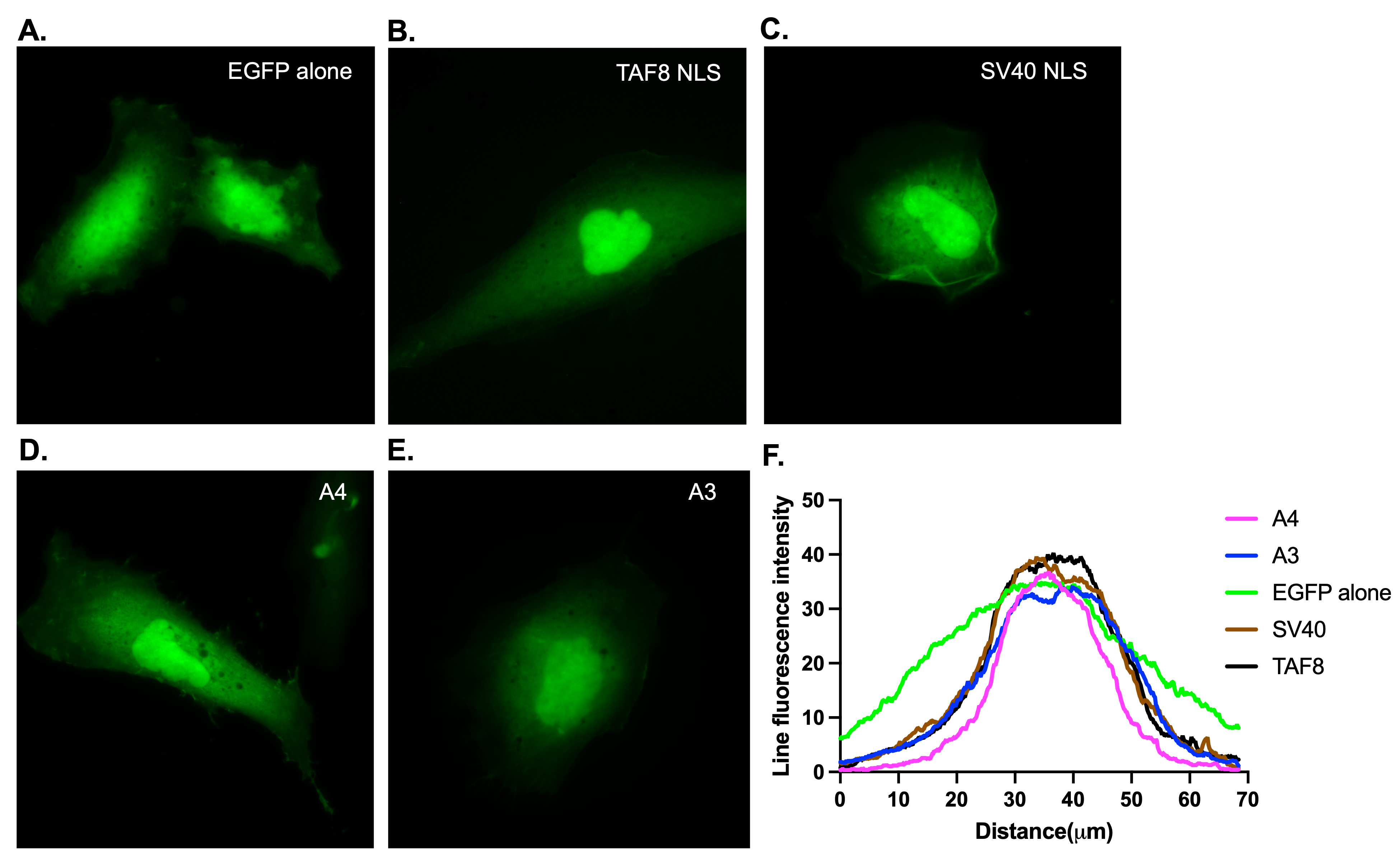
**

**
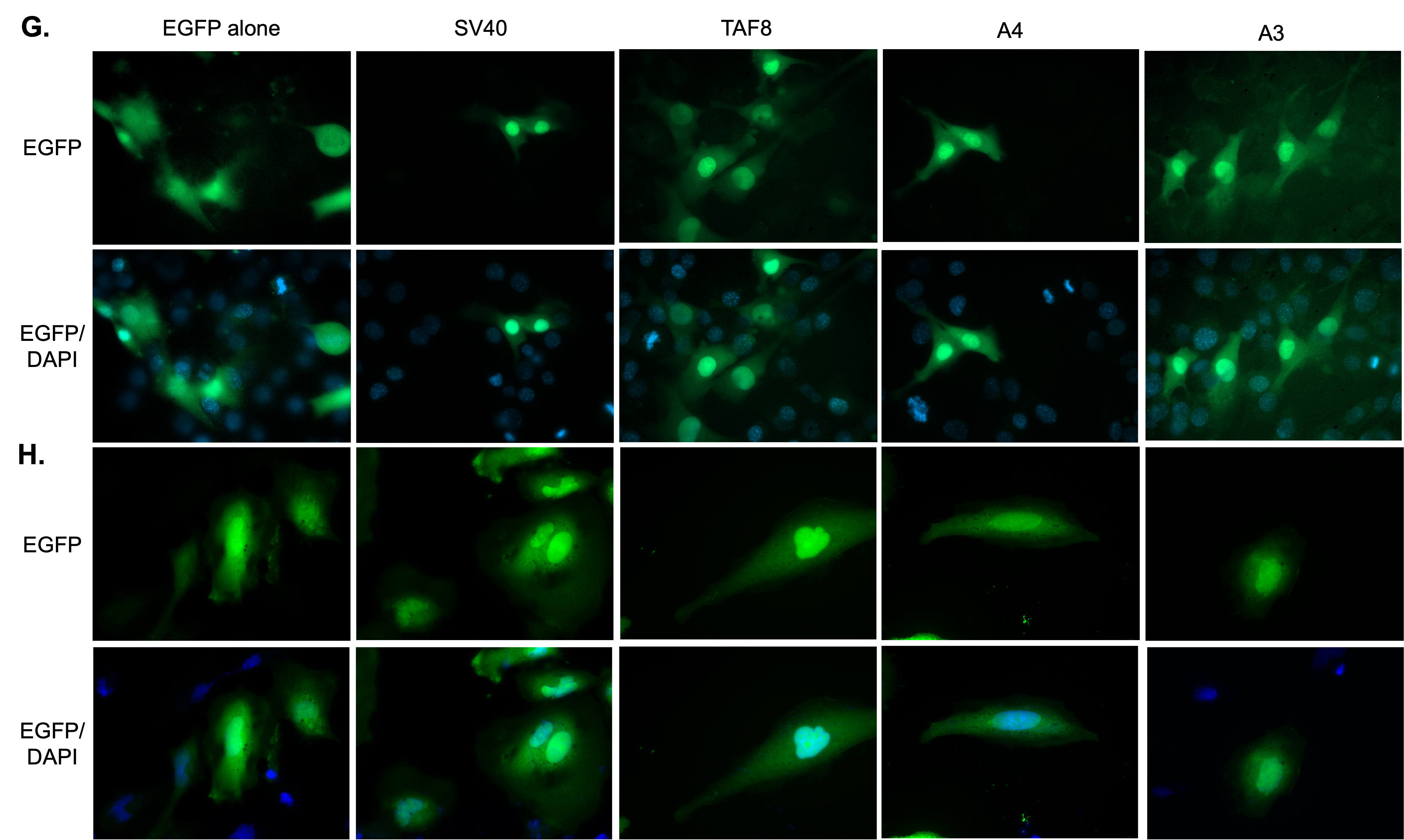
**

**
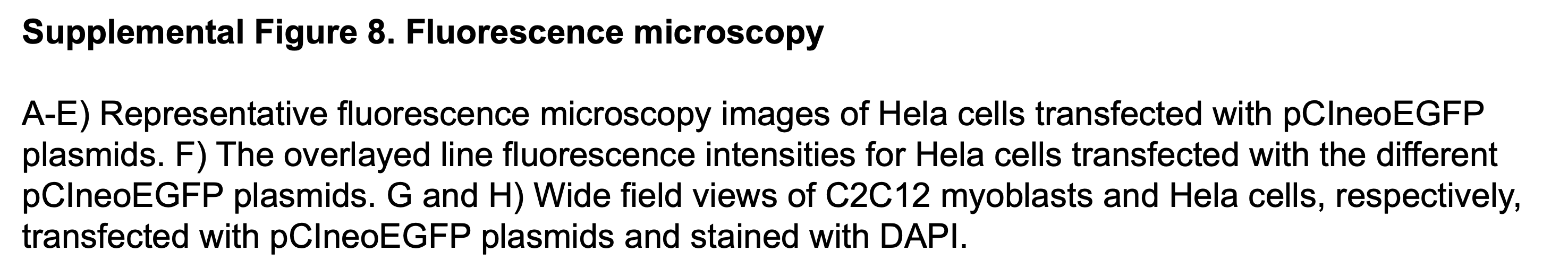
**

**SUPPLEMENTAL METHODS**

**Analog synthesis**

Fmoc-protected amino acids, diisopropylethylamine (DIPEA), 2-(1H-7-Azabenzotriazol-1-yl)-1,1,3,3-tetramethyl uranium hexafluorophosphate methanaminium (HATU) and trifluoroacetic acid (TFA) were purchased from Chem-impex international (Wood Dale, IL). The 2-chlorotrityl chloride resin was purchase from Matrix Innovation (Québec city, Québec, Canada). Triisopropylene (TIPS) and ethanedithiol (EDT) were obtained from Sigma-Aldrich corp (St. Louis, MO). Dimethylformamide (DMF), isopropanol (IPA) and dichloromethane (DCM) were purchased from VWR (Québec, Canada). Piperidine was obtained from A&C Chemicals (Québec, Canada). Ultra-high performance liquid chromatography (UPLC)-mass spectrometry analyses were performed with a Waters (Milford, MA) AQUITY H-class – SQD2 mass detector and PDA eλ UV-visible detector on a BEH, C18, 1.7 µm, 2.1 x 50 mm column. Purifications were performed on a Waters preparative UPLC system consisting of injector 2707, pump 2535, and detector 2489, with an ACE C18 250 x 21.2 mm, 5 µm column (Canadian Life Science, Ontario, Canada). For analytical UPLC, water and acetonitrile with 0.1% formic acid were used. For preparative UPLC, water plus 0.1% TFA, and pure acetonitrile were used. Peptide syntheses were performed on Symphony-X automated peptide synthesizer from Protein Technologies (Tucson, AZ) following manufacturer’s recommendations. The solid phase was solid phase Fmoc-Arg(Pbf)-2-Cl-Trtiyl resin, which was treated with a solution of Fmoc-Arg(Pbf)-OH or Fmoc-Glu(t-Bu)-OH (0.6 mmol) and diisopropylethylamine (1.2 mmol) in 30 mL of DCM for 1.5 hours. 2 mL of a 1:1 mixture of MeOH and diisopropylethylamine were added and agitated for 5 minutes. The resin was washed with DCM and IPA in alternance 5 times and dried overnight.

Analogs were synthesized using an automated Symphony-X Peptide Synthesizer, at 100 µmol scale. Fmoc groups deprotection was achieved using 20% piperidine in DMF. Couplings were performed using 5 eq of amino acids, activated with HATU and DIPEA (1:2 molar ratio in relation to the amino acid) for 30 min. The final deprotection was performed manually using 50% piperidine in DMF for 15 min and resin were washed using DMF x2, DCM x3, and IPA. The analogs were cleaved from their solid support using a mixture of TFA/water/TIPS/EDT (92.5/2.5/2.5/2.5) (4 mL for 200 mg of resin) for 3 h. Crude analogs were precipitated in chilled diethyl ether, centrifuged, and allowed to dry prior to reverse phase preparative UPLC purification. Final analogs were characterized using mass spectrometry and UPLC.

**Bacterial cell transformation, protein expression, purification and characterization**

Escherichia coli BL21 (DE3) strains transformed with this plasmid were cultured at 37°C in LB medium supplemented with 50 µg/mL kanamycin until reaching an optical density at 600 nm of 0.6, followed by induction with 0.5 mM isopropyl β–D–1–thiogalactopyranoside (IPTG) and a 3 h incubation at 30°C. After harvesting and washing the cells, they were lysed by sonication, and the lysate was removed by centrifugation. The soluble fraction was subjected to His-tag affinity chromatography using a 1 mL HisTRAP column (Cytiva, Marlborough, MA), pre-equilibrated with buffer A (25 mM Tris pH 7.5, 150 mM NaCl, 1 mM MgCl_2_, 20 mM imidazole, 2 mM dithiothreitol [DTT]), supplemented with a protease inhibitor cocktail and 0.25 mg/ml lysozyme (Calbiochem, Oakville, Canada). The bound proteins were washed with 10 column volumes (cv) of Buffer A, followed by a final wash with buffer A supplemented with 1 M NaCl (5 cv). The bound importin-ɑ was eluted through a 10 cv imidazole gradient ranging from 100 mM to 500 mM in Buffer A. The eluted fractions were then passed through a 1 mL HiTRAP Q HP column (Cytiva) and subjected to elution with a 10 cv NaCl gradient ranging from 125 to 1500 mM in buffer B (20 mM Tris pH 8.0, 125 mM NaCl, 2 mM DTT). Retained fractions were concentrated using a Vivaspin 30 kDa molecular weight cut-off (Sartorius, Goettingen, Germany) and further purified by size exclusion chromatography (SEC) using a Superdex200 HR 10/30 column (Cytiva) in buffer B. The monomeric form of importin-ɑ was retained, concentrated, flash-frozen in liquid nitrogen, and stored at -80°C. Immediate purification was evaluated by SDS-PAGE to determine the purity of the preparation. The impact of aggregate formation was evaluated over time using Superdex200 Increase 5/150 (Cytiva).

For crystallography studies, E. coli BL21 cells transformed with the pET30a-importin-ɑ plasmid were grown similarly and induced with 1 mM IPTG. After harvest, the cells were lysed by a French press, and the lysate removed as described above. The soluble fraction was applied to a pre-equilibrated gravity Ni–NTA column (Econo-Column; Bio-Rad, Hercules, CA) packed with HisPur Ni-NTA resin (Thermo Fisher Scientific, Waltham, MA). The bound proteins were washed successively with buffer C (20 mM Tris pH 8.2, 500 mM NaCl, 1 mM MgCl_2_, 50 mM imidazole, 0.9 mM DTT) and buffer D (20 mM Tris pH 8.2, 1 M NaCl, 500 mM MgCl_2_, 200 mM imidazole, 0.9 mM DTT), then eluted with buffer D. The His-tag was removed with bovine thrombin (Prolytix, Essex Junction, VT), and the mixture was dialyzed against buffer E (20 mM Tris pH 8.2, 100 mM NaCl, 2 mM DTT). The dialyzed and concentrated protein was loaded onto a HiLoadTM 16/600 SuperdexTM 200 column (Cytiva) pre-equilibrated with buffer F (10 mM Tris pH 7.78, 100 mM NaCl, 2 mM DTT). Fractions corresponding to the monomeric form of importin-ɑ were collected and concentrated to ~ 20 mg/mL, flash-frozen in liquid nitrogen, and stored at -80°C. All purification steps for crystallography were conducted at 4°C.

**Diffraction dataset collection and structure determination**

Diffraction datasets were collected at the Canadian Light Source (CLS) using beamline CMCF-ID with an Eager X 9M detector. The distance to the detector was 223.2 mm in all datasets apart from the mImpɑ2 with the A4 dataset where the distance was 262.8 mm. Data collection was performed at 100 °K, and a total of 720 images were collected with 0.25° oscillation range and 0.02 seconds exposure time per image at 0.9537 Å wavelength, for each dataset. For structure determination and refinement, all datasets were merged and scaled using the HKL2000 suite and phased using molecular replacement in Phaser with the previously solved structure of the TAF8 NLS-human importin-ɑ complex (PDB: 4WV6) as the search template [4-6]. The final structures were modelled and refined in Coot and Refmac5, respectively [7, 8]. 5% of the data were excluded for the R_free_ calculation.

**CD experiments**

We first fitted the denaturation curve of the apo-mImpα2 two the two-state unfolding model as described in Naud *et al*., [9] using a ΔC_p_°,_u_ of 1 cal·mol-1·k-1. This procedure allows for the determination of the T°, the ΔH°_u_(T°). With these values, the stability curve (ΔG°_u_(T)) of the apo-mImpα2 was established. The denaturation curves of the NLS:Impα2 pair were fitted accordingly, and the resulting stability curves were used to obtain the ΔΔG°u (ΔG°_u_ (NLS:Impα2) – (ΔG°_u_(mImpα2) at 25 and 37°C (Supplementary Table 1). A positive ΔΔG°u indicates a stabilization of mImpα2 from the Gibbs standard binding free energy of the NLS. This ΔΔG°u is also proportional, albeit not a measure, of the (relative) affinities of the NLS for the major and minor binding sites. Thermal denaturation values have previously been utilized when the midpoint exhibits measurable spectral increases during NLS binding relative to mImpɑ2 alone [10].

**Model fitting for binding affinities**

A two-binding site and Hill fitting model were also performed by using the following equation in GraphPad. The experiments were repeated three times and the calculated affinity values were reported as the mean equilibrium K_D_ ± standard deviation.

One binding site equation [11]:

$Y= \frac{\left( E_{t}{+L}_{t}{+K}_{D} \right)- \sqrt{{(E_{t}{+L}_{t}{+K}_{D})}^{2}- 4E_{t}L_{t}}}{2E_{t}}$

Where L_t_ is the total ligand concentration; protein concentration is E_t_; K_D_ is the dissociation constant or binding affinity.

Two binding site equation [11]:

$Y= \frac{\frac{L}{K_{D_{1}}} + \frac{2L^{2}}{K_{D_{1}}K_{D_{2}}}}{2 ( 1+ \frac{L}{K_{D_{1}}}+ \frac{L^{2}}{K_{D_{1}}K_{D_{2}}} )}$

Where $K_{D_{1}}$and $K_{D_{2}}$are the dissociation constants for binding site 1 and 2.

Hill equation [11]:

$Y= \frac{L^{n}}{{L^{n} +K}_{D}}$

Where L is known, and cooperativity is explicitly reported as the Hill coefficient (*n*) explicitly as K_D_ value.

**Plasmids and cloning method for confocal microscopy**

The empty pCIneoEGFP expression vector (Addgene, Cat#46949) was used as the backbone for all constructs. NLSs from TAF8 and SV40 were inserted downstream of the EGFP coding sequence. The analogs A3 (PEKKPKIEE) and A4 (PVKKEKIDE) were incorporated using the Gibson Assembly method. Plasmid backbone was amplified using Q5 High-Fidelity DNA Polymerase (New England Biolabs, Cat#M0491S). The following primers were used to introduce analog A3: Forward-F1: GAAGAAGCCCAAGATCGAGGAGTAAGCGGCCGCTTCCCTTTAG and Reverse-R3: CTCCTTCTTCACCGGCTTGTACAGCTCGTCCAT. The following primers were used to introduce analog A4: Forward-F1: TGAAGAAGGAGAAGATCGACGAGTAAGCGGCCGCTTCCCTTTAG and Reverse-R3: CTCCTTCTTCACCGGCTTGTACAGCTCGTCCAT. Oligonucleotides were synthesized by Thermo Fisher Scientific. PCR fragments were gel-purified using the E.Z.N.A. Gel Extraction kit (Omega Bio-tek, Cat#D2500-01), and assembly performed using the NEBuilder HIFI DNA Assembly Cloning kit (New England Biolabs, Cat#E5520S). All constructs were verified by Sanger sequencing. Maxipreps of plasmid DNA were performed using the PureLink HiPure Plasmid Maxiprep kit (Invitrogen, Cat#K210007).

**Plasmid transfection method for confocal microscopy**

Hela cells (ATCC, RRID: CVCL 0030) and C2C12 myoblasts (ATCC, RRID: CVCL 0188) were cultured in DMEM (Wisent, Cat#319-005-CL) supplemented with 15% FBS, 100 U/mL penicillin, and 100 µg/mL streptomycin. Cells were maintained at 37 ℃ in a humidified incubator with 5% CO_2_. Transfections were performed using Lipofectamine 2000 Transfection Reagent (Invitrogen, Cat#11668019) with 1 µg plasmid DNA per well according to the manufacturer’s instructions. Twenty-four hours post-transfection (transfection efficiency ~50% in Hela cells and ~10% in C2C12 myoblasts) underwent confocal microscopy analysis. Hela cells exhibited ~10-20% cell death following transfections, whereas no detectable cytotoxicity was observed in C2C12 myoblasts.

Hela cells were also transfected using the method developed by Schwoebel et al [12]. Briefly, plasmid transfection was performed in energy-depleted cells where Hela cells were washed with PBS and then incubated in glucose-free DMEM (Wisent) containing 100 U/mL penicillin, and 100 µg/mL streptomycin and 10% FBS, including 10 mM sodium azide and 6 mM 2-deoxy-D-glucose for 20 min prior to transfections. Fluorescence microscopy studies were performed at 1, 4, 12, and 24 h post-transfection while maintaining cells in the energy-depleted media.

**Supplemental References**

1. Delfing, B.M., et al., *Binding of viral nuclear localization signal peptides to importin-alpha nuclear transport protein.* Biophys J, 2023. **122**(17): p. 3476-3488.

2. Conti, E. and J. Kuriyan, *Crystallographic analysis of the specific yet versatile recognition of distinct nuclear localization signals by karyopherin alpha.* Structure, 2000. **8**(3): p. 329-38.

3. Fontes, M.R., T. Teh, and B. Kobe, *Structural basis of recognition of monopartite and bipartite nuclear localization sequences by mammalian importin-alpha.* J Mol Biol, 2000. **297**(5): p. 1183-94.

4. McCoy, A.J., et al., *Phaser crystallographic software.* J Appl Crystallogr, 2007. **40**(Pt 4): p. 658-674.

5. Otwinowski, Z. and W. Minor, *Processing of X-ray diffraction data collected in oscillation mode.* Methods Enzymol, 1997. **276**: p. 307-26.

6. Trowitzsch, S., et al., *Cytoplasmic TAF2-TAF8-TAF10 complex provides evidence for nuclear holo-TFIID assembly from preformed submodules.* Nat Commun, 2015. **6**: p. 6011.

7. Emsley, P., et al., *Features and development of Coot.* Acta Crystallogr D Biol Crystallogr, 2010. **66**(Pt 4): p. 486-501.

8. Murshudov, G.N., et al., *REFMAC5 for the refinement of macromolecular crystal structures.* Acta Crystallogr D Biol Crystallogr, 2011. **67**(Pt 4): p. 355-67.

9. Naud, J.F., et al., *Structural and thermodynamical characterization of the complete p21 gene product of Max.* Biochemistry, 2005. **44**(38): p. 12746-58.

10. Rizzuti, B., J.L. Iovanna, and J.L. Neira, *Deciphering the Binding of the Nuclear Localization Sequence of Myc Protein to the Nuclear Carrier Importin alpha3.* Int J Mol Sci, 2022. **23**(23).

11. Lefurgy, S.T. and T.S. Leyh, *Analytical expressions for the homotropic binding of ligand to protein dimers and trimers.* Anal Biochem, 2012. **421**(2): p. 433-8.

12. Schwoebel, E.D., T.H. Ho, and M.S. Moore, *The mechanism of inhibition of Ran-dependent nuclear transport by cellular ATP depletion.* J Cell Biol, 2002. **157**(6): p. 963-74.
